# Risk factor control in relation to mortality and life expectancy among people with type 2 diabetes: results from 3 nationwide cohort studies

**DOI:** 10.1186/s40779-025-00674-4

**Published:** 2025-12-05

**Authors:** Zi-Xin Qiu, Frank Qian, Yan-Bo Zhang, Jun Liu, Ting-Ting Geng, Rui Li, Pang Yao, Oscar H. Franco, Eric B. Rimm, JoAnn E. Manson, An Pan, Mai-Geng Zhou, Kai Huang, Gang Liu

**Affiliations:** 1 Department of Nutrition and Food Hygiene, Hubei Key Laboratory of Food Nutrition and Safety, Ministry of Education Key Laboratory of Environment and Health, and State Key Laboratory of Environment Health (Incubating), School of Public Health, Tongji Medical College, Huazhong University of Science and Technology, Wuhan, 430030 China; 2 Section of Cardiovascular Medicine, Boston Medical Center and Boston University Chobanian & Avedisian School of Medicine, Boston, MA 02118 USA; 3 Department of Epidemiology and Population Health, Albert Einstein College of Medicine, Bronx, NY 10461 USA; 4 Nuffield Department of Population Health, University of Oxford, Oxford, OX3 7LF UK; 5 Department of Epidemiology and Biostatistics, School of Public Health, Tongji Medical College, Huazhong University of Science and Technology, Wuhan, 430030 China; 6 Julius Center for Health Sciences and Primary Care, University Medical Center Utrecht, Utrecht University, Utrecht, 3584 CX The Netherlands; 7 Department of Nutrition, Harvard T.H. Chan School of Public Health, Boston, MA 02115 USA; 8 Department of Epidemiology, Harvard T.H. Chan School of Public Health, Boston, MA 02115 USA; 9 Channing Division of Network Medicine, Department of Medicine, Brigham and Women’s Hospital and Harvard Medical School, Boston, MA 02115 USA; 10 Department of Medicine, Brigham and Women’s Hospital, Harvard Medical School, Harvard University, Boston, MA 02115 USA; 11 Center for Obesity and Diabetes Research, School of Public Health, Tongji Medical College, Huazhong University of Science and Technology, Wuhan, 430030 China; 12 National Center for Chronic and Noncommunicable Disease Control and Prevention, Chinese Center for Disease Control and Prevention, Beijing, 102206 China; 13 Department of Cardiovascular Diseases, Union Hospital, Tongji Medical College, Huazhong University of Science and Technology, Wuhan, 430030 China; 14 Clinical Center for Human Genomic Research, Union Hospital, Huazhong University of Science and Technology, Wuhan, 430030 China; 15 Department of Cardiometabolic Diseases, Remin Hospital, Wuhan University, Wuhan, 430060 China

**Keywords:** Type 2 diabetes (T2D), Risk factors, Mortality, Life expectancy, Prospective study

## Abstract

**Background:**

Type 2 diabetes (T2D) is a global epidemic that reduces life expectancy. Evidence is limited on the benefits of achieving multiple guideline-recommended targets and cross-country differences. This study aimed to quantify the associations of risk factor control with mortality and life expectancy among individuals with T2D using nationwide cohorts from China, the USA, and the UK.

**Methods:**

We included 46,351 adults with T2D at baseline from China Chronic Disease and Risk Factors Surveillance (CCDRFS; 2013, follow-up until 2021), USA National Health and Nutrition Examination Survey (USA NHANES; 1999–2018, follow-up until 2019), and UK Biobank (2006–2010, follow-up until 2022). Patients with T2D were matched to controls without T2D using propensity score matching based on key demographic factors. Cox regression estimated mortality associated with lifestyle and metabolic factors outside target ranges [physical inactivity, smoking, unhealthy diet, elevated hemoglobin A1c (HbA1c), dyslipidemia, high blood pressure].

**Results:**

Only a small proportion of participants achieved ≥ 5 combined targets: 16.0% in CCDRFS, 9.9% in USA NHANES, and 6.8% in UK Biobank. During 470,369 person-years of follow-up, 7650 deaths (16.5%) occurred among individuals with T2D, and 9349 deaths (10.2%) occurred among controls over 965,249 person-years. At age 50, individuals with ≤ 1 risk factor outside the target lived 6–9 years longer than those with ≥ 5, and their life expectancy was comparable to that of controls without T2D. The association was independent of genetic predisposition to shorter lifespan in the UK Biobank. Additionally, individuals with T2D who failed to achieve optimal metabolic control but maintained a healthy lifestyle had a longer life expectancy compared with those who achieved optimal metabolic control but had an unhealthy lifestyle across all cohorts, with life expectancy gains ranging from 1.5 to 3.4 years depending on sex and cohort. Among individuals with T2D, healthy lifestyle behaviors (physical activity, non-smoking, a healthy diet) and HbA1c control contributed most to gains in life expectancy. Variations in multiple risk factor control and their associations with all-cause mortality were observed across different population subgroups.

**Conclusions:**

Achievement of guideline targets for multiple risk factors was low among individuals with T2D in China, the USA, and the UK. Comprehensive management of multiple risk factors, particularly lifestyle factors, was associated with a substantial reduction in the life expectancy gap between those with and without T2D, underscoring the importance of guideline-based care and individualized management.

**Supplementary Information:**

The online version contains supplementary material available at 10.1186/s40779-025-00674-4.

## Background

Type 2 diabetes (T2D) continues to be a global public health issue [1]. In 2025, the estimated number of individuals living with diabetes was approximately 589 million worldwide, and China and the USA have become the epicenters of the diabetes epidemic [2, 3]. On average, diabetes will reduce life expectancy in people aged 40–60 years by 4–10 years and independently increase the risk of death from cardiovascular disease (CVD), renal disease, and cancer by 1.3–3.0-fold [4]. Given the persistently increased prevalence of T2D, it is imperative to take measures for prevention and treatment, thereby extending the life expectancy of people with T2D.

Integrated multifactorial prevention, combining pharmacologic management of cardiovascular risk factors with behavioral modification, has been associated with lower risks of CVD, cancer, and mortality in people with T2D [5, 6]. However, nationwide data on achieving the control targets for metabolic and lifestyle factors recommended by diabetes guidelines are relatively limited [7–10]. There is increasing interest in understanding the association of multiple risk factor control with life expectancy. Nonetheless, it is still unclear to what extent achieving guideline-recommended targets can reduce the excess risk of death and the loss of life-years in individuals with T2D. Importantly, there are non-negligible differences between the Chinese, European, and the USA populations in terms of environmental exposures, metabolic health, and social determinants, including access to preventative therapies. Whether the magnitude of the associations varies across countries also needs to be clarified to inform tailored interventions and health policies. Furthermore, whether multiple risk factor control could offset the risk of premature mortality in those with increased genetic susceptibility is also not well understood.

In addition, despite recent advancements in medical treatments of T2D (e.g., new oral hypoglycemic agents), which offer significant benefits in improving cardiometabolic risk factors and reducing disease outcomes, there remains considerable residual risk of macrovascular and microvascular disease [6, 11]. As the pandemic of unhealthy diet and physical inactivity continues to escalate worldwide [12], it is essential to consider the role of a healthy lifestyle in reducing health risks. However, most people with T2D tend to focus predominantly on pharmacological therapies to address clinical risk factors, with less attention paid to lifestyle behaviors [13, 14]. Thus, whether adopting a healthy lifestyle can compensate for suboptimal metabolic control or provide additional longevity benefits among those who have already achieved recommended metabolic targets remains unclear.

To fill these knowledge gaps, we used data from 3 nationwide cohorts from China, the USA, and the UK, and examined the excess mortality risk and life-years lost in individuals with T2D according to the number of cardiovascular risk factors outside evidence-based target ranges, compared with people without T2D. Additionally, we investigated the associations of lifestyle behaviors with mortality and life expectancy in people with T2D who had different degrees of metabolic risk factor control.

## Methods

### Study design and population

Three large prospective and nationwide cohorts were used in this study: 1) the China Chronic Disease and Risk Factors Surveillance (CCDRFS; participants enrolled in June 2013 to May 2014, follow-up until 2021); 2) 10 cycles of USA National Health and Nutrition Examination Survey (NHANES; participants enrolled between 1999 and 2018, follow-up until 2019); and 3) UK Biobank (participants recruited between March 2006 and July 2010, follow-up until 2022). Detailed study designs for these cohorts are provided in the Additional file 1: Methods. The CCDRFS, USA NHANES, and UK Biobank were approved by the ethical review committees of the Chinese Center for Disease Control and Prevention (CDC) and other participating institutes, the USA National Center for Health Statistics Research Ethics Review Board, and UK National Health Service (NHS) National Research Ethics Service, respectively. Written informed consent was obtained from all adult participants.

For the present analyses, we included adults and excluded participants who were pregnant at baseline, withdrew from the study, or had incomplete data on modifiable risk factors (Additional file 2: Fig. S1). Each patient with prevalent T2D was matched with 2 controls without T2D using propensity score matching with the nearest neighbor method, in order to minimize confounding and to ensure comparability between groups. Definitions of T2D in each cohort followed the American Diabetes Association (ADA) criteria [15], as detailed in the Additional file 1: Methods. The matching criteria included age, sex, and residence area in the CCDRFS; age, sex, race, and survey cycles in USA NHANES; and age, sex, and race in UK Biobank. Our final analytical sample included 46,351 patients with T2D (17,423 from CCDRFS, 5752 from USA NHANES, and 23,176 from the UK Biobank), and 91,645 controls without T2D (34,799 from CCDRFS, 10,506 from USA NHANES, and 46,340 from the UK Biobank) (Additional file 2: Fig. S1). The slight discrepancies in the expected matching ratios were attributed to the internal optimization algorithm of the “MatchIt” R package used [16].

### Definition of achievement of guideline-recommended targets

We selected 3 clinical and 3 lifestyle targets based on international diabetes management guidelines and strong epidemiological evidence linking them to mortality and cardiovascular outcomes in people with T2D [5, 17–19]. The clinical targets were hemoglobin A1c (HbA1c) < 7.0%, systolic blood pressure < 140 mmHg or < 130 mmHg in the presence of CVD, and triglycerides (TG) < 1.7 mmol/L and high-density lipoprotein-cholesterol (HDL-C) ≥ 1.29 mmol/L for women, or ≥ 1.03 mmol/L for men [17, 18], given that TG/HDL-C abnormalities are more characteristic of T2D [20]. In a sensitivity analysis, individualized HbA1c targets were used to define glycemic targets (Additional file 1: Methods), and low-density lipoprotein-cholesterol levels (< 2.6 mmol/L) were used to determine lipid targets. The 3 lifestyle targets included never smoking, regular physical activity, and a healthy diet. Regular physical activity was defined as compliance with current global health guidelines for physical activity (≥ 150 min of physical activity per week) [21]. According to a previous CCDRFS study, a healthy diet was defined as intake of fruits and vegetables ≥ 400 g on average per day and intake of red meat ≤ 100 g on average per day [22]. In the USA NHANES, dietary quality was derived from 24-h dietary recalls and evaluated using the healthy eating index (HEI)-2010 scores [23]. A healthy diet was defined as the HEI in the top third of the distribution. In the UK Biobank, we utilized the dietary index developed by Rutten-Jacobs et al. [24], aligning with specific dietary targets recommended by the American Heart Association. A healthy diet was defined as meeting the recommended amount of at least 5 of the food items. Detailed information on the assessment of these risk factors is provided in Additional file 1: Methods.

We assigned 1 point for each risk factor variable outside the target range and 0 points for each risk factor variable within the target range. The unweighted risk score was determined by adding up the scores of the 6 aforementioned risk factors and then stratifying as optimal (0–1), suboptimal (2–4), and poor (5–6) risk factor control. The weighted score was also generated by using the β coefficients of each risk factor with mortality obtained from Cox proportional hazard models. To ensure similar proportions of participants in each category when classifying the weighted risk score, the thresholds for the weighted score were determined based on the corresponding percentile distribution of the unweighted score.

### Assessments of deaths

Deaths from CVD were defined as International Classification of Diseases, 10th Revision (ICD-10) codes I00 to I09, I11, I13, I20 to I51, and I60 to I69. In CCDRFS, deaths were ascertained by linkage to China’s Disease Surveillance Points System through December 31, 2021. In the USA NHANES, deaths were obtained by linkage to the National Death Index through December 31, 2019. In the UK Biobank, deaths were obtained through death certificates held within the NHS Information Center (England and Wales) and the NHS Central Register (Scotland) to December 20, 2022.

### Statistical analysis

The analysis in CCDRFS and USA NHANES considered sample weights, strata, and primary sampling units to produce accurate national estimates. Sample characteristics are presented as mean ±  SEM in CCDRFS and USA NHANES and mean ± standard deviation (SD) in UK Biobank for normally distributed continuous variables, and as median (interquartile range) for continuous variables not following a normal distribution. Categorical variables are presented as *n* (%). Person-time was calculated as the interval between baseline and the date of death, or censoring, whichever occurred first. The Cox proportional hazards regression model was used to estimate hazard ratios (*HR*s) and 95% confidence intervals (CIs). Schoenfeld residuals were used to test the proportional hazards assumption, and no violation was observed. For the main analysis, we estimated CVD and all-cause mortality risk and life expectancy among participants with T2D, according to the number of risk factors outside the target range, as compared with the controls without T2D. The models were adjusted for age (continuous, years), sex (males or females), race (White or non-White) (except for CCDRFS due to all participants being of a single racial/ethnic group), area of residence (urban or rural) (only in CCDRFS due to urban–rural health disparities arising from rapid urbanization in China), education, economic status, diabetes duration (continuous, years) (not available in CCDRFS), history of CVD (yes or no) and cancer (yes or no). Because the definitions and availability of education and economic status varied by cohort, we applied cohort-specific definitions. Education level was categorized into 3 groups in each cohort, harmonized to represent low, medium, and high educational attainment; economic status was represented by household income in the CCDRFS, the poverty-income ratio in the USA NHANES, and the Townsend deprivation index in the UK Biobank. To test the robustness of our findings, we conducted several sensitivity analyses. First, we used the full non-T2D population as an alternative reference group. Second, to reduce the potential impact of reverse causation, we excluded participants who died within the first 2 years of follow-up. Third, we assessed the associations of a weighted risk score with mortality and life expectancy. Fourth, we further adjusted for major medication use (i.e., diabetes medications, antihypertensive drugs, and lipid-lowering drugs). Stratified analyses were conducted by age at diagnosis (≤ 50 years or > 50 years), diabetes medication use (none, pills only, or insulin in USA NHANES and UK Biobank; yes or no in CCDRFS), presence of complications (yes or no), and socioeconomic status (SES) (low or high). We further evaluated the association of lifestyle risk factors with all-cause mortality and life expectancy, stratifying by the degree of metabolic risk factor control (i.e., whether individuals have attained optimal levels of all 3 metabolic risk factors or not).

Assuming that the observed association is causal, we calculated the population-attributable fraction (PAF), which quantifies the percentage of mortality in the population that is attributable to a specific risk factor. Adjusted PAF was calculated with the standard formula [25]: $PAF={P}_{d} \times \frac{{HR}_{adj}-1}{{HR}_{adj}}$; where *P*_*d*_ is the proportion of cases exposed to the risk factor, *HR*_*adj*_ is the adjusted *HR*, estimated by the multivariable Cox proportional hazards regression model. Details of the methods used to estimate life expectancy are provided in the Additional file 1: Methods [26–28]. By applying Arriaga’s decomposition method [29], we estimated the cause-specific contributions (CVD and non-CVD) to the life expectancy difference between participants with 0–1 risk factors and those with 5–6 risk factors among patients with T2D. For genetic analysis, a short lifespan-genetic risk score (GRS) was calculated [30]. We examined the association of risk factor control and life expectancy stratified by the GRS. Further details for genetic analysis are provided in the Additional file 1: Methods. Missing data (all missing values < 5%) were coded as a missing indicator category for categorical variables and median values for continuous variables.

All statistical analyses were conducted using SAS version 9.4 (SAS Institute Inc., Cary, NC, USA) and R software version 4.2.1 (R Foundation for Statistical Computing, Vienna, Austria). All statistical tests were two-sided, and *P* < 0.05 was considered statistically significant. Monte Carlo simulation (parametric bootstrapping) with 1000 runs was used to calculate the CIs of the life expectancy estimation.

## Results

### Study participants

Baseline characteristics of participants from CCDRFS, USA NHANES, and UK Biobank are shown in Table 1. The CCDRFS cohort comprised 17,423 people with T2D and 34,799 controls without T2D (Table 1). The mean age of people with T2D was (52.4 ± 0.6) years, and 46.1% were male. The USA NHANES cohort comprised 5752 people with T2D and 10,506 controls without T2D. The mean age (mean  ±  SEM) of people with T2D was (58.9 ± 0.3) years, and 50.8% were male. The UK Biobank cohort comprised 23,176 people with T2D and 46,340 controls without T2D. The mean age of people with T2D was (59.4 ± 7.3) years, and 63.9% were male. In CCDRFS, 16.0% (weighted proportion; unweighted: 2758/17,423) of patients achieved ≥ 5 targets; the corresponding proportions were 9.9% (weighted proportion; unweighted: 511/5752) in USA NHANES and 6.8% (unweighted proportion; 1568/23,176) in UK Biobank (Additional file 2: Fig. S2). For lifestyle targets, 15.4% (weighted proportion; unweighted: 3158/17,423) of patients in the CCDRFS achieved all 3 targets, while the proportions were 9.0% (weighted proportion; unweighted: 495/5752) in USA NHANES and 6.2% (unweighted proportion; 1429/23,176) in UK Biobank (Additional file 2: Fig. S3). For 3 clinical metabolic targets, the proportions were 16.1% (weighted proportion; unweighted: 2489/17,423) in CCDRFS, 13.8% (weighted proportion; unweighted: 747/5752) in USA NHANES, and 10.0% (unweighted proportion; 2318/23,176) in UK Biobank (Additional file 2: Fig. S3). In the USA NHANES and UK Biobank, participants with higher SES had better control of metabolic and lifestyle risk factors (*P* < 0.001) (Additional file 2: Table S1). Between-cohort comparisons revealed that patients with T2D in CCDRFS were more likely to be physically active and never smokers and to meet HbA1c and lipid targets, but had a lower educational level. In the USA NHANES, a higher proportion of patients met blood pressure targets. Across all cohorts, participants with more risk factors outside target ranges tended to be older, male, and have CVD at baseline (Table 1).

**Table 1:** Baseline characteristics of individuals with T2D and matched controls

Vriables	CCDRFS					USA NHANES					UK Biobank				
	Individuals with T2D				Matched controls (*n* = 34,799)	Individuals with T2D				Matched controls *(n* = 10,506)	Individuals with T2D				Matched controls (*n* = 46,340)
	Overall (*n* = 17,423)	No. of risk factor variables outside the target range				Overall (*n* = 5752)	No. of risk factor variables outside the target range				Overall (*n* = 23,176)	No. of risk factor variables outside the target range			
		≤ 1 (*n* = 2758)	2–4 (*n* = 13,669)	≥ 5 (*n* = 996)			≤ 1 (*n* = 511)	2–4 (*n* = 4304)	≥ 5 (*n* = 937)			≤ 1 (*n* = 1568)	2–4 (*n* = 17,341)	≥ 5 (*n* = 4267)	
Age [years, mean ± SEM/SD]	52.4 ± 0.6	49.1 ± 0.9	53.0 ± 0.6	54.8 ± 0.8	53.8 ± 0.4	58.9 ± 0.3	58.4 ± 0.5	59.0 ± 0.3	59.2 ± 0.6	60.9 ± 0.2	59.4 ± 7.3	58.6 ± 7.9	59.3 ± 7.3	60.0 ± 6.7	59.3 ± 7.3
Male [*n* (%)]	8030 (46.1)	1040 (37.7)	6206 (45.4)	784 (78.7)	17,782 (51.1)	2920 (50.8)	252 (49.4)	2178 (50.6)	490 (52.3)	5148 (49.0)	14,805 (63.9)	768 (49.0)	11,029 (63.6)	3008 (70.5)	28,545 (61.6)
Residence area (Urban) [*n* (%)]	8823 (50.6)	1249 (45.3)	7067 (51.7)	507 (50.9)	17,121 (49.2)	–	–	–	–	–	–	–	–	–	–
Education (College or University degree) [*n* (%)]	1253 (7.2)	232 (8.4)	943 (6.9)	78 (7.8)	2088 (6.0)	2738 (47.6)	319 (62.4)	2087 (48.5)	332 (35.4)	5946 (56.6)	8380 (36.2)	739 (47.1)	6954 (40.1)	687 (16.1)	21,270 (45.9)
Economic status^†^	34,919 (19,840–59,186)	35,300 (19,731–58,874)	34,890 (19,838–58,987	29,824 (19,563–50,124)	29,999 (17,956–53,966)	2.7 ± 0.04	3.3 ± 0.1	2.7 ± 0.04	2.4 ± 0.07	3.1 ± 0.04	–0.6 ± 3.4	–0.7 ± 3.3	–0.6 ± 3.4	–0.4 ± 3.4	–1.4 ± 3.1
History of CVD [*n* (%)]	904 (5.2)	94 (3.4)	724 (5.3)	86 (8.6)	1079 (3.1)	1401 (24.4)	69 (13.6)	994 (23.1)	338 (36.1)	1502 (14.3)	3989 (17.2)	181 (11.6)	2861 (16.5)	947 (22.2)	3105 (6.7)
History of cancer [*n* (%)]	466 (2.7)	77 (2.8)	355 (2.6)	34 (3.4)	766 (2.2)	867 (15.1)	72 (14.1)	676 (15.7)	119 (12.7)	1628 (15.5)	1987 (8.6)	125 (8.0)	1474 (8.5)	388 (9.1)	3707 (8.0)
Ever smoker [*n* (%)]	6079 (34.9)	226 (8.2)	5085 (37.2)	768 (77.1)	–	3060 (53.2)	78 (15.2)	2182 (50.7)	800 (85.4)	–	12,739 (55.0)	174 (11.1)	8861 (51.1)	3704 (86.8)	–
Suboptimal physical activity [*n* (%)]	3364 (19.3)	130 (4.7)	2583 (18.9)	651 (65.4)	–	3346 (58.2)	84 (16.5)	2402 (55.8)	860 (91.8)	–	9842 (42.4)	78 (5.0)	6312 (36.4)	3452 (80.9)	–
Unhealthy diet [*n* (%)]	12,666 (72.7)	1001 (36.3)	10,703 (78.3)	962 (96.6)	–	3644 (63.3)	86 (16.78)	2686 (62.4)	872 (93.1)	–	18,691 (80.6)	588 (37.5)	13,977 (80.6)	4126 (96.7)	–
Duration of diabetes [years, median (IQR)]	–	–	–	–	–	3.2 (0.5, 10.4)	1.9 (0.5, 8.0)	2.9 (0.5, 9.9)	5.1 (0.5, 13.7)	–	5.4 (2.3, 10.3)	4.7 (1.9, 9.6)	5.2 (2.2, 10.0)	6.6 (3.1, 11.6)	–
Elevated HbA1c [*n* (%)]	6225 (35.7)	185 (6.7)	5194 (38.0)	846 (84.9)	–	2436 (42.3)	31 (6.0)	1631 (37.9)	774 (82.6)	–	9129 (39.4)	99 (6.3)	5723 (33.0)	3307 (77.5)	–
Dyslipidemia [*n* (%)]	10,576 (60.7)	502 (18.2)	9117 (66.7)	957 (96.1)	–	4148 (72.1)	111 (21.8)	3125 (72.6)	912 (97.3)	–	16,502 (71.2)	279 (17.8)	12,139 (70.0)	4084 (95.7)	–
Elevated blood pressure [*n* (%)]	8158 (46.8)	350 (12.7)	6917 (50.6)	891 (89.5)	–	1849 (32.1)	27 (5.2)	1192 (27.7)	630 (67.2)	–	11,284 (48.7)	168 (10.7)	7630 (44.0)	3486 (81.7)	–

Normally distributed continuous variables are described as mean ± SEM in the USA NHANES and CCDRFS, and as mean ± SD in the UK Biobank, and continuous variables without a normal distribution are described as median [interquartile range (IQR)]. Categorical variables are presented as *n* (%). ^†^The economic status was represented as annual household income (¥) in CCDRFS [median (IQR)], the family poverty to income ratio in USA NHANES (mean ± SD), and the Townsend deprivation index in UK Biobank (mean ± SD). *IQR* interquartile range, *T2D* type 2 diabetes, *CVD* cardiovascular disease, *CCDRFS* China Chronic Disease and Risk Factors Surveillance, *NHANES* National Health and Nutrition Examination Survey

### Combined modifiable risk factors and outcomes

Across the 3 cohorts, during 470,369 person-years of follow-up, 7650 deaths (16.5%) occurred among 46,351 individuals with T2D, and 9349 deaths (10.2%) occurred among 91,645 controls over 965,249 person-years. In the CCDRFS cohort, over a maximum follow-up duration of 9.4 years, 2076 individuals with T2D (11.9%) and 2974 controls (8.5%) died. In the USA NHANES cohort, during a maximum follow-up period of up to 20.8 years, there were 1614 deaths among participants with T2D (28.1%) and 2342 deaths among controls (22.3%). In the UK Biobank, after a maximum follow-up of 13.3 years, 3960 deaths occurred among individuals with T2D (17.1%), compared to 4033 deaths among controls (8.7%). Table 2 shows the adjusted *HR*s for CVD and all-cause mortality associated with the number of risk factors outside evidence-based target levels at baseline in patients with T2D compared with controls without T2D. Patients with T2D who had ≥ 5 risk factors outside the target range had more than twice the risk of all-cause mortality compared with controls without T2D, with *HR*s (95% CIs) of 2.12 (1.62–2.77) in CCDRFS, 2.31 (2.00–2.68) in USA NHANES, and 2.36 (2.21–2.54) in UK Biobank. By contrast, patients with T2D who had ≤ 1 risk factor outside the target range showed no significant difference in mortality risk compared with controls (CCDRFS: *HR* = 0.99, 95% CI 0.77–1.27; USA NHANES: *HR* = 0.83, 95% CI 0.63–1.09; UK Biobank: *HR* = 1.15, 95% CI 0.98–1.36). Similar results were also observed for CVD mortality, except in the UK Biobank study, where T2D patients with ≤ 1 risk factor still had a higher risk of CVD mortality than the control group [*HR* = 1.84 (95% CI 1.40–2.41)].

**Table 2:** Adjusted hazard ratios (*HR*s) for CVD mortality and life expectancy at age 50, according to the number of risk factor variables outside target ranges, among patients with T2D, as compared with controls without T2D

Cohorts	Subgroups	Person-year	CVD mortality		All-cause mortality		Life expectancy [years (95% CI)]	
			Death (*n*)	*HR* (95% CI)	Death (*n*)	*HR* (95% CI)	Male	Female
CCDRFS								
	Matched controls	303,972	1365	Reference	2974	Reference	28.5 (28.5–28.6)	33.1 (33.1–33.2)
	People with T2D							
	≤ 1 risk factor	24,078	80	0.85 (0.55–1.30)	212	0.99 (0.77–1.27)	28.6 (28.1–29.0)	33.2 (32.7–33.6)
	2–4 risk factors	117,105	756	1.46 (1.23–1.89)	1669	1.53 (1.35–1.73)	25.0 (24.8–25.2)	29.6 (29.4–29.7)
	≥ 5 risk factors	8232	106	2.38 (1.64–3.45)	195	2.12 (1.62–2.77)	22.4 (21.8–22.8)	27.0 (26.5–27.4)
USA NHANES								
	Matched controls	91,502	743	Reference	2342	Reference	32.0 (31.9–32.0)	35.2 (35.1–35.2)
	People with T2D							
	≤ 1 risk factor	3724	25	1.04 (0.63–1.71)	66	0.83 (0.63–1.09)	33.9 (33.4–34.2)	36.8 (36.3–37.1)
	2–4 risk factors	34,534	396	1.60 (1.38–1.87)	1147	1.46 (1.33–1.60)	28.3 (28.2–28.4)	31.8 (31.7–31.9)
	≥ 5 risk factors	7700	141	2.63 (2.11–3.28)	401	2.31 (2.00–2.68)	23.8 (23.6–24.0)	27.6 (27.4–27.9)
UK Biobank								
	Matched controls	569,775	884	Reference	4033	Reference	32.6 (32.6–32.7)	35.0 (35.0–35.1)
	People with T2D							
	≤ 1 risk factor	19,098	49	1.84 (1.40–2.41)	150	1.15 (0.98–1.36)	31.4 (31.1–31.6)	33.9 (33.7–34.1)
	2–4 risk factors	206,628	849	2.28 (2.08–2.51)	2773	1.71 (1.63–1.80)	27.9 (27.8–28.0)	30.7 (30.5–30.8)
	≥ 5 risk factors	49,270	377	3.39 (2.97–3.87)	1037	2.36 (2.21–2.54)	25.1 (25.0–25.2)	28.0 (27.9–28.1)

The *HR*s were adjusted for age, sex, race (not adjusted in CCDRFS), residence area (adjusted in CCDRFS only), education level, economic status, history of CVD and cancer, and diabetes duration (not adjusted in CCDRFS). *T2D* type 2 diabetes, *CVD* cardiovascular disease, *CCDRFS* China Chronic Disease and Risk Factors Surveillance, *NHANES* National Health and Nutrition Examination Survey

These associations were robust across multiple sensitivity analyses (Additional file 2: Tables S2-S6). Specifically, after excluding participants who died during the first 2 years, the *HR*s for all-cause mortality comparing individuals with ≥ 5 risk factors outside target ranges vs. controls ranged from 2.15 to 2.37 across cohorts (Additional file 2: Table S2). Similar results were observed when using weighted risk scores (*HR*s 2.20–2.48) (Additional file 2: Table S3), applying individualized glycemic and lipid targets (*HR*s 2.12–2.43) (Additional file 2: Table S4), using all individuals without T2D as the reference group (*HR*s 2.07–2.36) (Additional file 2: Table S5), or further adjusting for major medication use (*HR*s 1.84–2.04) (Additional file 2: Table S6). By contrast, patients with T2D who had ≤ 1 risk factor outside the target range generally showed no significant difference in all-cause mortality compared with controls across most sensitivity analyses, including exclusion of participants who died during the first 2 years (*HR*s 0.85–1.17) (Additional file 2: Table S2), use of weighted risk scores (*HR*s 0.88–0.98) (Additional file 2: Table S3), individualized glycemic and lipid targets (*HR*s 0.88–1.20) (Additional file 2: Table S4), alternative reference group (*HR*s 0.83–1.16) (Additional file 2: Table S5), or additional adjustment for major medication use (*HR*s 0.82–1.01) (Additional file 2: Table S6). Some heterogeneity in the associations was observed when analyses were stratified by age at diagnosis, diabetes medication, presence of complications, and SES, although tests for interaction did not reach statistical significance in most stratified analyses (Additional file 2: Table S7). Among individuals treated with insulin, a higher number of risk factors outside the target range tended to be associated with greater mortality risk, and a statistically significant interaction was observed in the UK Biobank (*P*_interaction_ = 0.01). Specifically, the *HR*s (95% CIs) for all-cause mortality comparing individuals with ≥ 5 risk factors outside target ranges vs. controls were 3.15 (2.74–3.63) in the insulin-treated group, 2.23 (2.00–2.48) in the group using pills, and 1.98 (1.75–2.24) in the untreated group (Additional file 2: Table S7).

We calculated the PAF to estimate the percentage of mortality in the study population that theoretically would not have occurred if all individuals had been in the low-risk category (≤ 1 risk factor outside the target range). The PAFs for all-cause mortality were 51.5% (95% CI 27.4–68.0) in CCDRFS, 60.0% (95% CI 43.8–71.9) in USA NHANES, and 50.2% (95% CI 40.8–58.0) in UK Biobank. For CVD mortality, the PAFs were 61.4% (95% CI 33.7–77.5), 57.2% (95% CI 21.5–77.4), and 51.3% (95% CI 37.0–63.3), respectively.

**Fig. 1:**
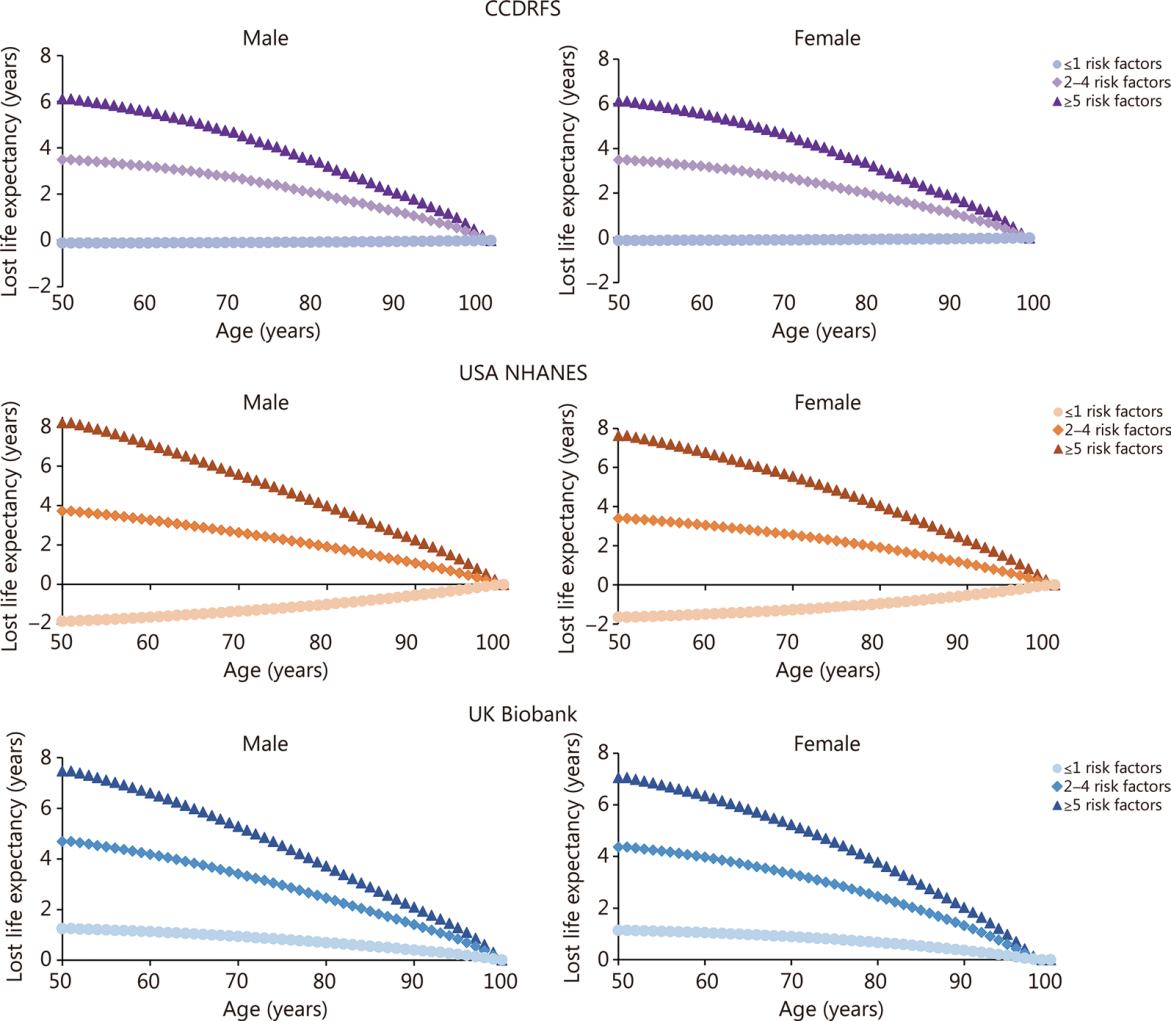
Estimated years of life lost according to the number of risk factor variables outside the target range among patients with T2D, as compared with controls without T2D. The *HR*s applied to estimate life expectancy were adjusted for age, sex, race (not adjusted in CCDRFS), residence area (adjusted in CCDRFS only), education level, economic status, history of CVD and cancer, and diabetes duration (not adjusted in CCDRFS). T2D type 2 diabetes, CVD cardiovascular disease, CCDRFS China Chronic Disease and Risk Factors Surveillance, NHANES National Health and Nutrition Examination Survey

In all 3 cohorts, individuals with T2D having ≥ 5 risk factors outside the target range had a life expectancy of 6.1–8.2 years less at age 50, compared with controls without T2D (Fig. 1). The differences in life expectancy for people with T2D having ≤ 1 risk factor outside the target range were between 1.2 and 1.9 years compared with controls without T2D. When comparing with people with T2D who had ≥ 5 risk factors, people with T2D who had ≤ 1 risk factor had 6, 9, and 6 years longer life expectancy on average in CCDRFS, USA NHANES, and UK Biobank, respectively (Fig. 1). On average, 60%, 30%, and 30% of the gained life expectancy at age 50 years from having ≤ 1 risk factor were attributable to reduced CVD deaths among people with T2D, in CCDRFS, USA NHANES, and UK Biobank, respectively (Additional file 2: Fig. S4). The life expectancy at age 50 years is also shown in Table 2.

### Lifestyle risk factors, metabolic risk factors, and outcomes

Adopting a healthier lifestyle (having 2–3 low-risk lifestyle factors) was associated with lower risk of CVD and all-cause mortality and longer life expectancy, even among patients who had optimal levels of all 3 metabolic risk factors. Specifically, the multivariable-adjusted *HR*s (95% CIs) ranged from 0.28 (0.14–0.57) to 0.62 (0.38–1.03) for CVD mortality and from 0.53 (0.35–0.80) to 0.79 (0.67–0.93) for all-cause mortality, resulting in an estimated 3.1–6.2 years longer life expectancy (Table 3). In addition, when optimal metabolic risk factor control cannot be achieved, adopting a healthier lifestyle was also associated with lower mortality risk and longer life expectancy, with *HRs* (95% CIs) ranging from 0.74 (0.65–0.84) to 0.79 (0.61–1.01) for CVD mortality and from 0.65 (0.60–0.71) to 0.72 (0.60–0.86) for all-cause mortality, and 2.0–4.2 years longer life expectancy (Table 3). Notably, individuals with T2D who failed to achieve optimal metabolic control but maintained a healthy lifestyle had a longer life expectancy compared with those who achieved optimal metabolic control but had an unhealthy lifestyle across all cohorts, with life expectancy gains ranging from 1.5 to 3.4 years depending on sex and cohort (Table 3).

**Table 3:** Adjusted hazard ratios (*HR*s) for CVD mortality and life expectancy at age 50, according to the number of low-risk lifestyle factors, among patients with T2D, stratified by metabolic risk factor control^*^

Cohorts	Subgroups	Person-year	CVD mortality		All-cause mortality		Life expectancy [years (95% CI)]	
			Death (*n*)	*HR* (95% CI)	Death (*n*)	*HR* (95% CI)	Male	Female
CCDRFS								
	Optimal metabolic risk factor control							
	0–1 low-risk lifestyle factors	7622	45	Reference	119	Reference	23.4 (22.6–24.0)	26.3 (26.9–28.3)
	2–3 low-risk lifestyle factors	13,879	38	0.28 (0.14–0.57)	121	0.53 (0.35–0.80)	29.0 (26.2–31.8)	33.4 (30.6–36.2)
	Suboptimal metabolic risk factor control							
	0–1 low-risk lifestyle factors	39,947	346	Reference	762	Reference	24.0 (22.7–25.2)	28.4 (26.9–29.7)
	2–3 low-risk lifestyle factors	87,968	513	0.79 (0.61–1.01)	1074	0.72 (0.60–0.86)	26.0 (24.7–27.9)	30.5 (28.0–32.6)
USA NHANES								
	Optimal metabolic risk factor control							
	0–1 low-risk lifestyle factors	2428	50	Reference	96	Reference	26.0 (23.7–28.3)	29.5 (27.3–31.5)
	2–3 low-risk lifestyle factors	1966	19	0.43 (0.21–0.87)	47	0.54 (0.38–0.76)	32.2 (29.2–34.9)	35.1 (32.6–37.6)
	Suboptimal metabolic risk factor control							
	0–1 low-risk lifestyle factors	25,774	334	Reference	1050	Reference	25.2 (24.5–25.9)	28.8 (28.1–29.5)
	2–3 low-risk lifestyle factors	15,789	159	0.77 (0.61–0.97)	421	0.67 (0.58–0.78)	29.4 (29.0–29.7)	32.6 (32.3–32.9)
UK Biobank								
	Optimal metabolic risk factor control							
	0–1 low-risk lifestyle factors	15,412	53	Reference	189	Reference	28.4 (28.2–28.6)	31.2 (31.0–31.3)
	2–3 low-risk lifestyle factors	12,505	23	0.62 (0.38–1.03)	94	0.79 (0.67–0.93)	31.7 (31.5–31.9)	34.3 (34.1–34.4)
	Suboptimal metabolic risk factor control							
	0–1 low-risk lifestyle factors	157,328	873	Reference	2733	Reference	26.7 (26.6–26.7)	29.5 (29.4–29.5)
	2–3 low-risk lifestyle factors	89,750	326	0.74 (0.65–0.84)	944	0.65 (0.60–0.71)	30.0 (29.5–30.5)	32.7 (32.2–33.1)

*The term “optimal metabolic risk factor control” refers to individuals who have achieved optimal levels of all three metabolic risk factors, namely blood glucose, blood pressure, and lipid levels. Conversely, “suboptimal risk factor control” is defined as individuals who have not achieved optimal levels of all these metabolic risk factors. *HR*s were adjusted for age, sex, race (not adjusted in CCDRFS), residence area (adjusted in CCDRFS only), education level, economic status, history of CVD and cancer, and diabetes duration (not adjusted in CCDRFS). *T2D* type 2 diabetes, *CVD* cardiovascular disease, *CCDRFS* China Chronic Disease and Risk Factors Surveillance, *NHANES* National Health and Nutrition Examination Survey

### Individual modifiable risk factors and outcomes

The associations between individual risk factors and life expectancy are presented in Additional file 2: Table S8. Among patients with T2D, achieving targets for never smoking, regular physical activity, healthy diet, and glucose control was associated with gains in life expectancy of 1.2–2.9, 2.6–4.1, 0.7–2.0, and 1.9–3.5 years, respectively. In contrast, achieving target levels for blood pressure and blood lipids was associated with smaller or uncertain gains (ranging from –0.1 to 0.9 years).

### Modifiable risk factors, GRSs, and outcomes

For genetic analysis among participants with T2D in the UK Biobank, according to Model 2, the adjusted *HR* of all-cause mortality risk for the high genetic risk group was 1.06 (95% CI 1.01–1.13) compared with those in the low genetic risk group (Additional file 2: Table S9). Better risk factor control at age 50 years was associated with longer life expectancy in both low and high genetic risk groups (Additional file 2: Fig. S5). No significant multiplicative and additive interactions between risk factor control and genetic susceptibility to a short lifespan were found (*P*_interaction_ > 0.05). It was estimated that, compared with people with T2D having ≥ 5 risk factors, those having ≤ 1 risk factor gained 6.3–6.6 life-years in the low genetic risk group and 5.6–5.9 life-years in the high genetic risk group (Additional file 2: Fig. S5).

## Discussion

Leveraging 3 nationwide cohorts from China, the USA, and the UK, we estimated the associations of 6 cardiovascular risk factors at target with mortality risk and life expectancy in people with T2D. We found that the life expectancy of people with T2D was comparable to those without T2D of similar age, sex, and race, if most risk factors were under control, including smoking, physical activity, diet, blood glucose, blood pressure, and blood lipids. Additionally, adherence to a healthy lifestyle was associated with lower mortality risk and longer life expectancy, irrespective of whether target levels of HbA1c, blood pressure, and lipids were achieved. The association was independent of genetic predisposition to shorter lifespan in the UK Biobank. Moreover, variations in multiple risk factor control and their associations with all-cause mortality were observed across different population subgroups, highlighting that individualized approaches based on patients’ profiles may further improve health outcomes in people with T2D.

Evidence from clinical trials on the effects of multifactorial interventions for patients with T2D remains inconclusive. For instance, in the Steno-2 Study among 160 patients with T2D and microalbuminuria, behavior modification (i.e., improvements in diet quality and physical activity) together with use of medications (i.e., antihypertensive drugs and aspirin) led to a significant reduction in CVD events [31] and a 7.9-year gain in life expectancy after 7.8 years of follow-up [9]. The Look AHEAD trial, involving 5145 adults with T2D, reported significant improvements in body weight, HbA1c, blood pressure, and HDL-C after 4 years of follow-up [32, 33] but showed no significant reduction in CVD and all-cause mortality after an intensive lifestyle intervention (i.e., calorie restriction and increased physical activity) [34]. For these intervention studies among people with diabetes, inconsistent findings may be at least partly attributable to factors such as limited intervention or follow-up duration, or minimal differences in lifestyle modification between groups. Most observational studies have focused on lifestyle behaviors, with limited data assessing the combined impact of multiple risk factor modifications on survival in people with diabetes. One study using the United Kingdom Prospective Diabetes Study Outcomes Model projected that control of major risk factors (smoking, blood pressure, HbA1c, and total cholesterol: HDL-C ratio) could extend life expectancy by up to 21 years in people with diabetes [8]. Another analysis of UK Biobank data by using the area under the survival curve to calculate residual life expectancy found that adherence to multiple healthy lifestyle factors (non-smoking, healthy diet, optimal BMI, physical activity, and control of HbA1c, blood pressure, and total cholesterol) was associated with an approximately 6-year gain in life expectancy among people with diabetes [10]. Variations in these estimates are likely due to differences in risk factor definitions, participant characteristics, study designs (observational vs. interventional), and statistical methods used to estimate life expectancy.

In the present study, we conducted an analysis of the associations of modifiable factors with mortality and life expectancy using the life table method based on data from 3 large nationwide cohorts and contemporary national-level mortality rates [26–28]. This study provides novel evidence quantifying the association between multiple risk factor control and life expectancy in the Chinese population with T2D. We found that the estimated life expectancy at age 50 years for individuals with ≤ 1 risk factor outside the target range was, on average, 6 years longer than that of those with ≥ 5 risk factors outside the target range in China. Similar estimates were observed in the USA NHANES and UK Biobank, with corresponding values of approximately 9 and 6 years, respectively. The modest differences in effect sizes across cohorts may be explained by variations in genetic background, dietary habits, lifestyle factors, disease burden patterns, and healthcare systems [35, 36]. Differences in data collection methods and study designs across the cohorts from different countries may also contribute to these observed variations. Despite these differences, the consistent finding that multiple controlled risk factors are associated with substantially longer life expectancy underscores the importance of attaining the current guideline-recommended target levels of risk factors in people with T2D. This observed association may be mediated by common biological pathways, including improved endothelial function, reduced oxidative stress, attenuation of chronic low-grade inflammation, and preserved cardiomyocyte function, that contribute to lower cardiovascular mortality in T2D [37].

Additionally, in people with T2D, a healthy lifestyle was associated with a 21–47% lower mortality risk and 3.1–6.2 years longer life expectancy, even with optimal metabolic risk factor control. When optimal control was not achieved, a healthy lifestyle still conferred a 28–35% lower risk and 2.0–4.2 years longer life expectancy. Our findings highlight the significance of prioritizing lifestyle modifications among individuals with T2D, in addition to controlling clinical risk factors, which will often rely significantly on pharmacological agents. By modifying behavioral factors, multiple risks that commonly accompany diabetes (e.g., those related to cardiovascular and psychological factors) can be comprehensively reduced [38]. Despite these findings, lifestyle modifications are frequently overlooked in clinical practice for a variety of reasons, such as time constraints and lack of training [14, 39]. For example, national data from the USA indicated that only 56.3% of patients with diabetes seen by a clinician in 2010 received physical activity counseling [40]. Changing the built environment to promote smoking cessation, regular physical activity, and healthier dietary patterns will be key to ensuring more widespread and equitable access to addressing these lifestyle-related factors. Given the well-established cardiovascular benefits of novel antidiabetic agents, such as glucagon-like peptide-1 receptor agonists, future studies are warranted to assess whether these therapies can modify the effects of comprehensive risk factor control on mortality in T2D.

When examining the risk factors individually, we consistently found that the top factors associated with greater life expectancy were healthy lifestyle behaviors and controlled HbA1c levels in people with T2D. The potential years of life gained were smaller when achieving target levels for blood pressure or blood lipids. Similarly, a previous study in people with T2D also showed that blood pressure and blood lipids had generally smaller effects on mortality compared with smoking, physical activity, and HbA1c [5]. The associations of blood lipids and blood pressure with mortality risk can be complex, especially in people with diabetes, with growing studies suggesting a U-shaped relationship [41–43]. However, strong evidence supports the use of statins [44] and certain antihypertensive drugs [37] for primary and secondary CVD prevention in people with T2D, and the U-shaped pattern should not discourage their use.

Consistent with previous studies [45, 46], we found cardiovascular risk factor control generally remained poor across cohorts. The low rates of lifestyle and metabolic target achievement among individuals with T2D likely reflect a combination of behavioral, healthcare, and structural barriers. These include an obesogenic environment, poor adherence to healthy behaviors, rising obesity prevalence, low awareness of diabetes, and barriers related to healthcare access [47]. Additionally, broader contextual factors, such as aging populations, urbanization, psychosocial stress, and environmental pollution, may further hinder effective risk factor control [47]. We also noted that lower SES was associated with poorer risk factor control, particularly in the UK and USA cohorts. Comprehensive actions are needed to address both individual behaviors and system-level support to improve diabetes management and extend longevity, particularly by reducing SES-related disparities and incorporating personalized treatment strategies tailored to individual metabolic profiles, genetic background, and demographics to enhance intervention effectiveness.

The strengths of our study included using 3 prospective cohorts from different countries with various races/ethnicities. These cohorts were selected for their national representativeness (CCDRFS and USA NHANES) or nationwide coverage (UK Biobank), rigorous study designs, and large sample sizes. The results were largely consistent in these 3 cohorts, indicating the generalizability of our findings. The current study also had potential limitations. First, lifestyle behaviors were self-reported and may, in turn, lead to misclassification, although it would be generally expected to bias the associations towards the null in a prospectively designed study. Second, the lack of repeated measurements limits our ability to account for long-term patterns or changes in lifestyle and metabolic factors, and future studies with repeated assessments are needed. Third, aligned with the previous study [6], we matched the T2D and non-T2D groups on age, sex, and race, which are major drivers of mortality outcomes. While this matching enhances comparability, it may potentially limit the generalizability of the findings; however, sensitivity analyses using the general population as a reference group yielded similar results. Fourth, while both the CCDRFS and USA NHANES are nationally representative, the UK Biobank is restricted to adults aged 40–69 years at baseline, and selection bias may be present. Fifth, we did not include lifestyle factors other than smoking, physical activity, and diet due to inconsistent evidence and insufficient data on other factors, such as alcohol and obesity. Future studies incorporating other detailed lifestyle data are warranted. Finally, because of the observational nature of the studies, residual and unmeasured confounding cannot be fully ruled out, and causal relationships cannot be established.

## Conclusions

Based on 3 nationwide cohorts from China, the USA, and the UK, this study provides evidence that life-years lost in T2D could be substantially reduced through comprehensive risk factor control. Moreover, individuals with T2D who met most lifestyle and metabolic targets exhibit a life expectancy comparable to those without T2D. In addition, given that the overall risk factor management was poor, achieving further longevity gains will require more intensive and comprehensive management in patients with T2D, including both optimized medical interventions and prioritized lifestyle modifications. Variations in multiple risk factor control and their associations with all-cause mortality across population subgroups further suggest that individualized approaches could help improve survival.

## Supplementary Information

**Additional file 1. Methods.**

**Additional file 2. Table S1** Distribution of the number of risk factors outside target ranges by socioeconomic status in CCDRFS, USA NHANES, and UK Biobank. **Table S2** Adjusted hazard ratios (*HR*s) for mortality by numbers of risk factors outside target ranges among patients with type 2 diabetes (T2D) vs. controls without T2D, excluding participants who died during the first 2 years of follow-up. **Table S3** Adjusted hazard ratios (*HR*s) for mortality by weighted risk scores among patients with type 2 diabetes (T2D) vs. controls without T2D. **Table S4** Adjusted hazard ratios (*HR*s) for mortality by numbers of risk factors outside target ranges*, among patients with type 2 diabetes (T2D) vs. controls without T2D. **Table S5** Adjusted hazard ratios (*HR*s) for mortality by numbers of risk factors outside target ranges among patients with type 2 diabetes (T2D) vs. all individuals without T2D. **Table S6** Adjusted hazard ratios (HRs) for mortality by number of risk factors outside target ranges among patients with type 2 diabetes (T2D) vs. controls without T2D, with further adjustment for medication use. **Table S7** Stratified analysis of adjusted hazard ratios (*HR*s) for all-cause mortality by the number of risk factors outside target ranges among patients with type 2 diabetes (T2D) vs. controls without T2D. **Table S8** Adjusted hazard ratios (*HR*s) and years of life gained at age 50 by individual risk factors among patients with type 2 diabetes. **Table S9** Adjusted hazard ratios (*HR*s) for all-cause mortality according to genetic risk among participants with type 2 diabetes in the UK Biobank. **Fig. S1** Flow chart of study participants. **Fig. S2** Distribution of the number of risk factors within the target range. **Fig. S3** Proportion of participants achieving all lifestyle targets (**a**) and all clinical metabolic targets (**b**). **Fig. S4** Estimated years of life lost from having ≥ 5 risk factors vs. ≤ 1 risk factors attributable to reduced death from CVD and other causes among people with T2D. **Fig. S5** Estimated life expectancy at age 50 according to the number of risk factors outside the target ranges in people with T2D stratified by genetic risk in the UK Biobank.

## Data Availability

Data from the USA NHANES and the UK Biobank (Application No. 109546) are available to any legitimate scientific research on application. Data from the CCDRFS are available from the corresponding authors upon reasonable request.

## Abbreviations

ADA: American Diabetes Association
BMI: Body mass index
CCDRFS: China Chronic Disease and Risk Factors Surveillance
CDC: Center for disease control and prevention
CVD: Cardiovascular disease
GRS: Genetic risk score
HbA1c: Hemoglobin A1c
HDL: High-density lipoprotein
HEI: Healthy eating index
NHANES: National Health and Nutrition Examination Survey
NHS: National Health Service
PAF: Population-attributable fraction
SES: Socioeconomic status
T2D: Type 2 diabetes
TG: Triglycerides
