# Supplementary material for: Risk factor control in relation to mortality and life expectancy among people with type 2 diabetes: results from 3 nationwide cohort studies
Source: Mil Med Res. 2025 Dec 5;12:89. doi: 10.1186/s40779-025-00674-4 (PMC12679727; doi:10.1186/s40779-025-00674-4)
Supplement: Supplementary file 1 — Additional file 1. Methods. [file 40779_2025_674_MOESM1_ESM.pdf]

# Methods

## China Chronic Disease and Risk Factors Surveillance (CCDRFS)

The CDRFS is a nationally representative, cross-sectional, and prospective study organized by the Chinese Center for Disease Control and Prevention (CDC) [1]. It was designed to monitor the epidemiology of noncommunicable diseases and their risk factors in China. In the 2013 survey, which was conducted from June 2013 to May 2014, 179,347 participants were enrolled from 298 across mainland China, and the response rate was 93.4%. All 298 sites were randomly selected with an iterative method involving multistage stratification to ensure that these sites covered the representative geographical areas of all 31 provinces, autonomous regions, and municipalities in mainland China. The multistage sampling and quality control process were reported previously [1]. Trained interviewers from local centers for the CDC carried out the face-to-face interviews, physical measurements, biochemical sample collection, and sample pretreatment. For most subjects, interviews were conducted during home visits. Physical measurement and biochemical sample collections were conducted at a community health center.

## USA National Health and Nutrition Examination Survey (NHANES)

The NHANES is an ongoing program of study that provides population estimates related to nutrition and health of adults and children in the USA [2]. The survey used a stratified, multistage probability design to recruit a representative sample of the USA population. Continuous NHANES data have been released by the National Center for Health Statistics (NCHS) every 2 years for public use since 1999. In this analysis, we used 10 cycles between 1999 and 2018. Data were obtained via personal structured interviews at home, health examinations at a mobile examination center, and specimen analyses in the laboratory

## UK Biobank

The UK Biobank is a very large, population-based prospective study, established to allow detailed investigations of the genetic, lifestyle, and environmental determinants of the diseases of middle and old age [3]. Between March 2006 and July 2010, half a million participants aged 40 – 69 years who lived within 25 miles of 1 of the 22 assessment centers located throughout England, Wales, and Scotland were recruited into the UK Biobank. To ensure that a representative sample of the population

was selected, a centralized booking system and helpline coordinated this process and ensured an even throughput of volunteers attending assessment centers. Each assessment center visit lasted approximately 90 min, during which participants underwent questionnaire administration, physical measurements, and the collection of biological samples.

**Ascertainment of type 2 diabetes (T2D)**

In CCDRFS, prevalent cases of T2D were identified by at least one of the following criteria: 1) self-reported prior diagnosis by a health care professional; 2) plasma glucose ≥ 7 mmol/L (126 mg/dl with fasting time ≥ 8 h); 3) postprandial 2-hour plasma glucose ≥ 11.1 mmol/L (200 mg/dl from an oral glucose tolerance test); 4) hemoglobin A1c (HbA1c) ≥ 6.5% (48 mmol/L) [4].

In USA NHANES, prevalent cases of T2D were identified by at least one of the following criteria: 1) history of doctor diagnosis of diabetes; 2) use of insulin or oral hypoglycemic drugs; 3) serum glucose ≥ 7 mmol/L (126 mg/dl with fasting time ≥ 8 h); 4) postprandial 2-hour plasma glucose ≥ 11.1 mmol/L (200 mg/dl from an oral glucose tolerance test); 5) HbA1c ≥ 6.5% (48 mmol/L) [4].

In UK Biobank, prevalent cases of T2D were identified by at least one of the following criteria: 1) history of doctor diagnosis of diabetes; 2) use of insulin or hypoglycemic drugs; 3) hospital diagnosis of diabetes that occurred before date of recruitment [International Classification of Diseases, Tenth Revision (ICD-10) codes, E11]; 4) serum glucose ≥ 7 mmol/L (126 mg/dl with fasting time ≥ 8 h), or serum glucose ≥ 11.1 mmol/L (200 mg/dl without fasting time limitation); 5) HbA1c ≥ 6.5% (48 mmol/L) [4].

**Assessment of main exposures**

***Medical examination data***

In CCDRFS, after 5 min rest, 3 consecutive measures of blood pressure were assessed using the Omron digital blood pressure monitor (Omron, Dalian, China) with 1 min between each measure. The mean of these 3 measurements was assigned as the participants' blood pressure. Triglycerides (TG) and high-density lipoprotein-cholesterol (HDL-C) were measured using enzyme colorimetry (Roche COBAS C501 automatic biochemical analyzer). HbA1c was measured by high-performance liquid chromatography analysis. Details of the laboratory procedures and quality control methods were described elsewhere [1].

In the USA NHANES, blood pressure was obtained by having participants rest quietly for 5 min,

after which 3 measurements were taken. The mean of these 3 measurements was assigned as the participants' blood pressure. TG was assayed using enzymatic reactions. HDL-C was analyzed through a modified traditional multistep precipitation reaction. HbA1c was measured by high-performance liquid chromatography analysis. The laboratory procedures and quality control methods can be found in the USA NHANES's online protocol (https://wwwn.cdc.gov/nchs/nhanes/ContinuousNhanes/LabMethods.aspx?BeginYear=2003).

In the UK Biobank, after 5 min of seated rest, blood pressure was measured on 2 consecutive occasions with a 1-minute interval; the mean of the first and the second automated readings was adopted for the data analysis whenever available. TG was assayed using enzymatic analysis. HDL-C was analyzed using the enzyme-immunoassay method. HbA1c was measured by high-performance liquid chromatography analysis. The laboratory procedures and quality control methods can be found in the UK Biobank's online protocol (http://www.ukbiobank.ac.uk/).

***Smoking***

In CCDRFS, smoking status was obtained using the Global Adult Tobacco Survey questionnaire [5]. Ever smoker was defined as an adult who self-reported cigarette use in his or her lifetime.

In the USA NHANES, participants were asked about whether they had smoked over 100 cigarettes in life, and we defined those who had smoked over 100 cigarettes in life as ever smokers.

In the UK Biobank, participants were asked about their current tobacco smoking status, including yes (on most or all days), only occasionally, no, and prefer not to answer. Those who did not smoke on most or all days would be asked about the past tobacco smoking status, including smoked on most or all days, smoked occasionally, just tried once or twice, never smoked, or prefer not to answer. Those who reported that they smoked occasionally or just tried once or twice would be asked about whether they had smoked a total of at least 100 times in their lifetime. According to the information, participants who never smoked and those who previously smoked occasionally or just tried once or twice but did not smoke 100 times in their lifetime were categorized into the "never smoking" group, similar to the USA NHANES. Others with no missing information on smoking would be viewed as ever smokers.

***Physical activity***

In CCDRFS and UK Biobank, the Global Physical Activity Questionnaire (GPAQ) released by the World Health Organization (WHO) was used to collect data on physical activity [6]. Participants were asked about how many days per week they typically engaged in moderate to vigorous work,

transportation, and moderate to vigorous leisure time. For each category in which an answer of one or more days was given, the participant was subsequently asked the number of minutes typically spent on the activity per day. We summed the total minutes of moderate activity, vigorous activity (weighted by 2), and transportation as the total amount of physical activity.

In the USA NHANES, for physical activity, the questionnaire changed from a specific Physical Activity and Physical Fitness Questionnaire before the 2007 – 2008 cycle to the GPAQ thereafter. Both questionnaires assessed the duration of physical activity from different domains. Briefly, the former assessed minutes of physical activity during the past 30 d from the household, transportation, and moderate to vigorous leisure time; the latter measured minutes of physical activity in a typical week from moderate to vigorous work, transportation, and moderate to vigorous leisure time. We summed the total minutes of moderate activity, vigorous activity (weighted by 2), and transportation as the total amount of physical activity.

***Diet***

In CCDRFS, a semi-quantitative food frequency questionnaire was used to investigate the frequency and average intake of each food group (including vegetables, fruits, livestock, poultry, and fish) within the past 12 months. The daily consumption of vegetables and red meat in grams was calculated.

In the USA NHANES, dietary information was obtained through 24-hour dietary recalls, and an observation validation study showed that the 24-hour dietary recall could accurately estimate intakes of energy and macronutrients. The dietary interviews were conducted by trained investigators following the USA Department of Agriculture Automated Multiple-Pass Method for the 24-hour recall. First, investigators asked respondents about what they consumed yesterday, which could match foods from the Main Food List, which contained more than 2600 food items. Second, some specific categories of foods that were frequently forgotten, including fruits, vegetables, cheese, bread, sweets, snacks, nonalcoholic beverages, and alcoholic beverages, were additionally asked. Next, eating occasions and time, food descriptions, and food amounts of each food were obtained. Consumptions of food groups and nutrients were determined using the USA Department of Agriculture Nutrient Database for Dietary Studies and Food Patterns Equivalents Database. Dietary quality was evaluated through the Healthy Eating Index (HEI)-2010 scores [7]. The HEI-2010 was aligned with the 2010 Dietary Guidelines for Americans. Components and scoring standards of HEI-2010 are shown in **Table S1**.

**Table S1** Components and scoring standards of the healthy eating index-2010 score in the USA NHANES [1]

| Component | Maximum points | Standard for maximum score | Standard for the minimum score of zero |
|---|---|---|---|
| Adequacy components (higher score indicates higher consumption) | | | |
| Total fruits [2] | 5 | ≥ 0.7 cup equiv. per 1000 kcal | No fruits |
| Whole fruits [3] | 5 | ≥ 0.3 cup equiv. per 1000 kcal | No whole fruits |
| Total vegetables [4] | 5 | ≥ 0.9 cup equiv. per 1000 kcal | No vegetables |
| Greens and beans [4] | 5 | ≥ 0.1 cup equiv. per 1000 kcal | No dark green vegetables or legumes |
| Whole grains | 10 | ≥ 1.5 oz equiv. per 1000 kcal | No whole grains |
| Dairy [5] | 10 | ≥ 2.0 cup equiv. per 1000 kcal | No dairy |
| Total protein foods [6] | 5 | ≥ 2 oz equiv. per 1000 kcal | No protein foods |
| Seafood and plant proteins [6,7] | 5 | ≥ 0.5 oz equiv. per 1000 kcal | No seafood or plant proteins |
| Fatty acids [8] | 10 | (PUFAs + MUFAs)/SFAs ≥ 1.5 | (PUFAs + MUFAs)/SFAs ≤ 0.9 |
| Moderation components (higher score indicates lower consumption) | | | |
| Refined grains | 10 | ≤ 1.5 oz equiv. per 1000 kcal | ≥ 3.4 oz equiv. per 1000 kcal |
| Sodium | 10 | ≤ 1.1 gram per 1000 kcal | ≥ 1.7 grams per 1000 kcal |
| Added sugars | 10 | 0% of energy | ≥ 13.8% of energy |
| Saturated fats | 10 | ≤ 12.2% of energy | ≥ 18.2% of energy |

[1] Intakes between the minimum and maximum standards are scored proportionately. The total HEI score is the sum of the adequacy components (i.e., foods to eat more of for good health) and moderation components (i.e., foods to limit for good health). [2] Includes 100% fruit juice. [3] Includes all forms except juice. [4] Includes legumes (beans and peas). [5] Includes all milk products, such as fluid milk, yogurt, and cheese, and fortified soy beverages. [6] Includes legumes (beans and peas). [7] Includes seafood, nuts, seeds, soy products (other than beverages), and legumes (beans and peas). [8] Ratio of [polyunsaturated fatty acids (PUFAs) + monounsaturated fatty acids (MUFAs)]/saturated fatty acids (SFAs). *HEI* healthy eating index, *equiv.* equivalent

In the UK Biobank, according to a previous UK Biobank study [8], dietary quality was evaluated using more recent dietary recommendations for cardiovascular health, and details are shown in **Table S2**. Fruit intakes were evaluated according to daily consumption of fresh fruit (pieces) and dried fruit (pieces). Vegetable intakes were evaluated according to daily consumption of cooked vegetables (tablespoons) and salad/raw vegetables (tablespoons). Whole grain intakes were evaluated according to weekly consumptions of bread (slices, if the participant consumed whole-meal/whole grain bread) and cereal (bowls, if the participant consumed bran, oat, or Muesli). (Shell)Fish were evaluated according to the frequency of eating oily fish and non-oily fish. Dairy intakes were evaluated according to the frequency of cheese consumption and whether the participant consumed milk. Vegetable oils were evaluated according to the weekly consumption of bread (slices) if the participants consumed

Flora Pro-Active/Benecol, soft margarine, olive oil-based, polyunsaturated/sunflower oil-based, or other low/reduced fat spread (**Table S2**). Refined grain intakes were evaluated according to weekly consumption of bread (slices, if the participant consumed white, brown, or other bread) and cereal (bowls, if the participant consumed biscuit or others). Unprocessed meat consumptions were evaluated according to the frequency of consumption of poultry, beef, lamb/mutton, and pork, and whether the participants did not eat meat anymore (according to a question about age when last ate meat). Processed meat consumptions were evaluated according to the frequency of consumption and whether the participants did not eat it anymore (according to a question about the age they last ate meat). Sugar-sweetened beverage intakes were evaluated by a question: "Which of the following do you NEVER eat?" Those who chose sugar or foods/drinks containing sugar were regarded as never drinking sugar-sweetened beverages.

**Table S2** Definition of each component of a healthy diet score in the UK Biobank

| **Components** | **Goal (1 point)** | **One serving equals** |
|---|---|---|
| Fruits | ≥ 3 servings/d | 1 piece of fresh fruit, 5 pieces of dried fruit |
| Vegetables (excluding potatoes) | ≥ 3 servings/d | 3 heaped tablespoons |
| Whole grains | ≥ 3 servings/d | 1 slice of whole-grain bread, 1 cup of whole-grain cereal |
| Vegetable oil | ≥ 2 servings/d | Vegetable oil-based spread (Flora Pro-Active/Benecol, soft(tub) margarine, olive oil-based spread, or polyunsaturated/sunflower oil-based spread) in combination with eating at least 2 slices of bread |
| Fish | ≥ 2 servings/d | Once/week |
| Dairy | ≥ 2 servings/d | 1 cup/d of any type of milk, 1 piece of cheese |
| Refined grains | ≥ 2 servings/d | 1 slice of bread, 1 bowl of cereal |
| Unprocessed meats | ≥ 2 servings/d | Once/week (including poultry, beef, lamb, and pork) |
| Processed meats | ≤ 1 servings/week | Once/week |
| Sugar-sweetened beverages | Don't drink | Only 0 servings were possible here |

## Definition of individualized HbA1c targets

Less than 6.5% for young adults aged 18 – 44 years without cardiovascular diseases (CVDs), < 7.0% for both young adults with CVDs and middle-aged adults aged 45 – 64 years without CVDs, < 8.0% for both middle-aged adults and older adults aged 65 years or older with CVDs, and < 7.5% for older adults without CVDs [9].

**Statistical methods used for estimating life expectancy**

We combined 3 pieces of information to estimate the life expectancy among people with T2D according to individual or combined risk-factor variables outside target ranges as compared with people without T2D (henceforth "exposure groups"). (1) Population-based prevalences of each exposure group estimated from the CCDRFS, USA NHANES, or UK Biobank. (2) Population-based all-cause mortality rates in 2019 from the GBD website, USA CDC WONDER database, or the UK's Office for National Statistics. (3) Hazard ratios (*HR*s) for all-cause mortality among patients with T2D according to individual or combined risk-factor variables, as compared with the controls without T2D derived from the CCDRFS, USA NHANES, or UK Biobank.

The life tables for each of the exposure groups in males and females, separately, were built on the estimated population mortality rates in each exposure group. We estimated reductions in life expectancy as differences in expectation of life at any given age between any 2 life tables compared.

Population-based prevalence of exposure groups by sex and 5-year age intervals came from the CCDRFS, USA NHANES, or UK Biobank. The sex- and age-specific prevalences of exposure groups were assumed to be constant in each 5-year interval.

Population all-cause mortality rates per 100,000 during the year 2019 were obtained for the Chinese population by sex and 5-year age groups from the GBD website (http://ghdx.healthdata.org/gbd-results-tool). Population all-cause mortality rates per 100,000 were obtained in 1-year age-groups for the USA population during the year 2019 from the CDC WONDER online database (https://wonder.cdc.gov/ucd-icd10.html). The corresponding values for the UK population were obtained from the Office for National Statistics (https://www.ons.gov.uk/peoplepopulationandcommunity/birthsdeathsandmarriages/lifeexpectancies/datasets/singleyearlifetablesuk1980to2018). Because CDC WONDER only provide mortality rates up to age 84 years old, but we desired to estimate the overall population survival curves until 100 years, we used a Poisson regression model with both linear and quadratic terms for the midpoints of single-year age groups minus age 40.5 years to extrapolate the mortality rates for each single year of age after 84 years [10] (**Fig. S1**).

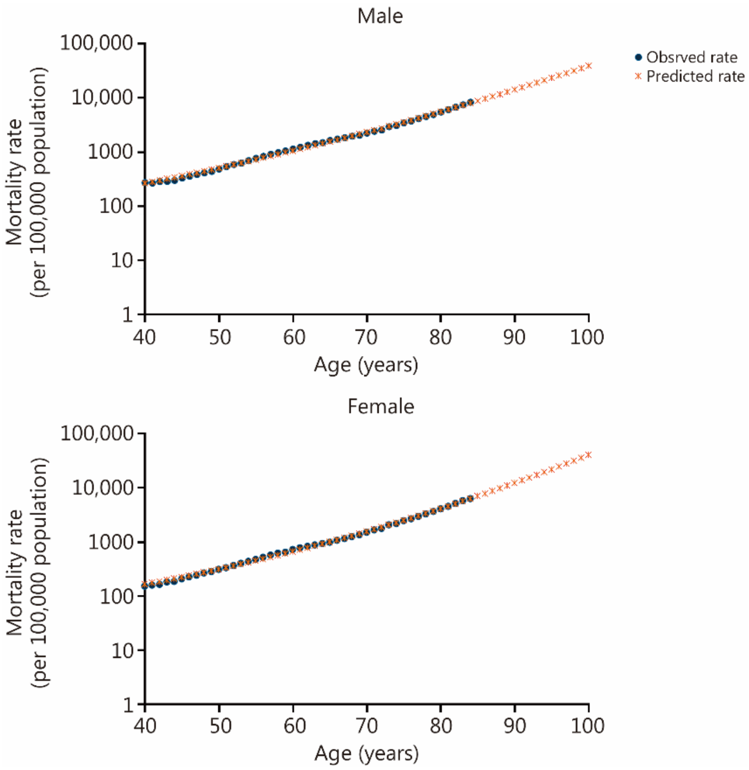


**Fig. S1** Observed and predicted rate of USA population all-cause mortality rates of 2019

*HR*s for mortality associated with risk factor levels as compared with the matched controls, assumed to be constant across age groups, were estimated from the CCDRFS, USA NHANES, or UK Biobank. We fitted Cox proportional hazards regression models, with adjustment for age, sex, race (not adjusted in CCDRFS), area of residence (adjusted in CCDRFS only), education, income, diabetes duration (not adjusted in CCDRFS because this information was not available), history of CVD, and cancer.

We used an algebraic transformation to infer the age-specific mortality rates appropriate for the reference group $IRa0$ by sex as [11]:

$$IR_{a0} = \frac{IR_a}{P_{a0} + \sum_{j=1}^{j=n} P_{aj} \times HR_j}$$

Where $IR_a$ is the population mortality rate for age group a; $P$aj is the age-specific prevalence of exposure groups; $HR_j$ is the *HR* for comparison of group j vs. reference group (j = 0). The age-specific mortality rates for each non-reference group were then inferred by multiplying the age-specific mortality rate for the reference group $IRa0$ and the $HR_{aj}$.

Finally, based on the sex- and age-specific mortality rate, life tables were built for each exposure group. Survival probability was set as 1 at the age of 40. The probability of surviving between ages x

and x + 1 was then estimated based on the probability of dying (mortality rate) between ages x and x + 1, assuming that the survivor function declines linearly between ages x and x + 1 [12, 13]. For the last open-ended age interval, the probability of surviving was set to 0. The life expectancy at any given age was derived by dividing the total person-years that would be lived beyond age x by the number of persons who survived to that age interval [12].

**Statistical methods used for genetic analysis**

The short lifespan-genetic risk score (GRS) was calculated from a large longevity GWAS reported by Deelen et al. [14]. Participants were categorized into low vs. high genetic risk groups based on the median of the weighted GRSs. Three independent single-nucleotide polymorphisms (SNPs) showed significant genome-wide association with parental longevity in the UK Biobank (90th percentile cases vs. all controls) [14]. The GRS was calculated using the previously reported weighted method: weighted GRS = (β1 × SNP1 + β2 × SNP2 + β3 × SNP3) × (3/sum of the 3β coefficients), with higher scores indicating greater genetic susceptibility to shorter lifespan. Participants were categorized into low vs. high genetic risk groups based on the median of the weighted GRSs. For genetic analyses, we further adjusted for the genotype measurement batch and genetic principal components. We examined the potential multiplicative interaction between risk factor control and genetic susceptibility using the likelihood ratio test comparing models with and without a cross-product term. Additive interaction was evaluated by calculating the relative excess risk due to the interaction, as described previously [15]. We also assessed the association between risk factor control and life expectancy in patients with T2D who had low and high genetic risk.

**References**


1. Zhang M, Wang L, Wu J, Huang Z, Zhao Z, Zhang X, et al. Data resource profile: China Chronic Disease and Risk Factor Surveillance (CCDRFS). Int J Epidemiol. 2022;51(2):e1-e8.
2. Centers for Disease Control and Prevention (CDC). National Health and Nutrition Examination Survey (NHANES) Analytic and Reporting Guidelines. Natl Cent Health Stat. 2000. https://www.researchgate.net/publication/2414208_National_Health_and_Nutrition_Examination_Survey_NHANES_Analytic_and_Reporting_Guidelines. Accessed 5 Nov 2024.
3. Sudlow C, Gallacher J, Allen N, Beral V, Burton P, Danesh J, et al. UK Biobank: an open access resource for identifying the causes of a wide range of complex diseases of middle and old age.

PLoS Med. 2015;12(3):e1001779.

4. American Diabetes Association. Classification and diagnosis of diabetes: standards of medical care in diabetes—2019. Diabetes Care. 2019;42(Suppl 1):S13-S28.
5. World Health Organization. Tobacco questions for surveys: a subset of key questions from the Global Adult Tobacco Survey (GATS): Global Tobacco Surveillance System. Geneva: World Health Organization. 2011. https://cdn.who.int/media/docs/default-source/ncds/ncd-surveillance/tqs-gats234dafb7-fcb0-4ba2-b8c7-d46de28b7e9f.pdf. Accessed 5 Nov 2024.
6. Craig CL, Marshall AL, Sjöström M, Bauman AE, Booth ML, Ainsworth BE, et al. International Physical Activity Questionnaire: 12-country reliability and validity. Med Sci Sports Exerc. 2003;35(8):1381-95.
7. Guenther PM, Kirkpatrick SI, Reedy J, Krebs-Smith SM, Buckman DW, Dodd KW, et al. The healthy eating index-2010 is a valid and reliable measure of diet quality according to the 2010 Dietary Guidelines for Americans. J Nutr. 2014;144(3):399-407.
8. Rutten-Jacobs LC, Larsson SC, Malik R, Rannikmäe K, Sudlow CL, Dichgans M, et al. Genetic risk, incident stroke, and the benefits of adhering to a healthy lifestyle: cohort study of 306,473 UK Biobank participants. BMJ. 2018;363:k4168.
9. American Diabetes Association. Glycemic targets: standards of medical care in diabetes—2022. Diabetes Care. 2022;45(Suppl 1):S83-S96.
10. Li Y, Pan A, Wang DD, Liu X, Dhana K, Franco OH, et al. Impact of healthy lifestyle factors on life expectancies in the US population. Circulation. 2018;138(4):345-55.
11. Woloshin S, Schwartz LM, Welch HG. The risk of death by age, sex, and smoking status in the United States: putting health risks in context. J Natl Cancer Inst. 2008;100(12):845-53.
12. Chiang CL. The life table and its applications. Malabar (FL): R.E. Krieger Publishing Company; 1984.
13. Arias E, Xu J. United States life tables, 2019. Natl Vital Stat Rep. 2022;70(19):1-59.
14. Deelen J, Evans DS, Arking DE, Tesi N, Nygaard M, Liu X, et al. A meta-analysis of genome-wide association studies identifies multiple longevity genes. Nat Commun. 2019;10(1):3669.
15. Li R, Chambless L. Test for additive interaction in proportional hazards models. Ann Epidemiol. 2007;17(3):227-36.
