# Supplementary material for: Risk factor control in relation to mortality and life expectancy among people with type 2 diabetes: results from 3 nationwide cohort studies
Source: Mil Med Res. 2025 Dec 5;12:89. doi: 10.1186/s40779-025-00674-4 (PMC12679727; doi:10.1186/s40779-025-00674-4)
Supplement: Supplementary file 2 — Additional file 2. Table S1 Distribution of the number of risk factors outside target ranges by socioeconomic status in CCDRFS, USA NHANES, and UK Biobank. Table S2 Adjusted hazard ratios (HRs) for mortality by numbers of risk factors outside target ranges among patients with type 2 diabetes (T2D) vs. controls without T2D, excluding participants who died during the first 2 years of follow-up. Table S3 Adjusted hazard ratios (HRs) for mortality by weighted risk scores among patients with type 2 diabetes (T2D) vs. controls without T2D. Table S4 Adjusted hazard ratios (HRs) for mortality by numbers of risk factors outside target ranges*, among patients with type 2 diabetes (T2D) vs. controls without T2D. Table S5 Adjusted hazard ratios (HRs) for mortality by numbers of risk factors outside target ranges among patients with type 2 diabetes (T2D) vs. all individuals without T2D. Table S6 Adjusted hazard ratios (HRs) for mortality by number of risk factors outside target ranges among patients with type 2 diabetes (T2D) vs. controls without T2D, with further adjustment for medication use. Table S7 Stratified analysis of adjusted hazard ratios (HRs) for all-cause mortality by the number of risk factors outside target ranges among patients with type 2 diabetes (T2D) vs. controls without T2D. Table S8 Adjusted hazard ratios (HRs) and years of life gained at age 50 by individual risk factors among patients with type 2 diabetes. Table S9 Adjusted hazard ratios (HRs) for all-cause mortality according to genetic risk among participants with type 2 diabetes in the UK Biobank. Fig. S1 Flow chart of study participants. Fig. S2 Distribution of the number of risk factors within the target range. Fig. S3 Proportion of participants achieving all lifestyle targets (a) and all clinical metabolic targets (b). Fig. S4 Estimated years of life lost from having ≥ 5 risk factors vs. ≤ 1 risk factors attributable to reduced death from CVD and other causes among people with T2D. Fig. S5 Estimated [file 40779_2025_674_MOESM2_ESM.pdf]

Table S1 Distribution of the number of risk factors outside target ranges by socioeconomic status in CCDRFS, USA NHANES, and UK Biobank (%)

**Stratified variable**	**≤ 1 risk factor**	**2 – 4 risk factors**	**≥ 5 risk factors**	***P*-value**
CCDRFS				
Household income				0.78
< 20,000 ¥	16.59	77.86	5.55	
≥ 20,000 ¥	15.43	78.40	6.17	
Education level				0.53
Primary school or below	15.80	78.84	5.36	
Secondary school/vocational	15.84	77.70	6.46	
College or university degree	18.74	75.31	5.95	
USA NHANES				
Family income-poverty ratio				< 0.001
Below median	7.14	74.94	17.92	
Above median	12.32	75.06	12.63	
Education level				< 0.001
Less than high school	5.00	72.73	22.26	
High school or equivalent	9.15	76.22	14.63	
College or above	12.87	76.23	10.89	
UK Biobank				
Townsend deprivation index				< 0.001
Below median	7.03	75.31	17.66	
Above median	6.38	74.14	19.48	
Education level				< 0.001
No formal qualifications	4.87	72.74	22.39	
O levels/GCSEs/CSEs or equivalent	6.45	74.10	19.44	
A levels or equivalent	6.68	75.63	17.69	
Higher education	7.92	75.89	16.19	

CCDRFS China Chronic Disease and Risk Factors Surveillance, *NHANES* National Health and Nutrition Examination Survey, *GCSEs* General Certificate of Secondary Education, *CSEs* Certificate of Secondary Education

Table S2 Adjusted hazard ratios (*HR*s) for mortality by numbers of risk factors outside target ranges among patients with T2D vs. controls without T2D, excluding participants who died during the first 2 years of follow-up

Cohorts	Subgroups	CVD mortality		All-cause mortality	
		Death (*n*)	*HR* (95% CI)	Death (*n*)	*HR* (95% CI)
CCDRFS					
	Matched controls	1222	Reference	2607	Reference
	People with T2D				
	≤ 1 risk factor	72	0.83 (0.55 – 1.27)	189	1.03 (0.99 – 1.35)
	2 – 4 risk factors	669	1.48 (1.11 – 1.98)	1437	1.53 (1.33 – 1.76)
	≥ 5 risk factors	85	2.25 (1.45 – 3.49)	167	2.15 (1.59 – 2.89)
USA NHANES					
	Matched controls	656	Reference	2070	Reference
	People with T2D				
	≤ 1 risk factor	22	1.16 (0.69 – 1.96)	54	0.85 (0.63 – 1.15)
	2 – 4 risk factors	345	1.68 (1.43 – 1.98)	983	1.47 (1.33 – 1.63)
	≥ 5 risk factors	121	2.76 (1.19 – 3.47)	341	2.37 (2.03 – 2.79)
UK Biobank					
	Matched controls	825	Reference	3764	Reference
	People with T2D				
	≤ 1 risk factor	48	1.71 (1.27 – 2.30)	142	1.17 (0.99 – 1.38)
	2 – 4 risk factors	792	2.26 (2.04 – 2.50)	2563	1.71 (1.62 – 1.80)
	≥ 5 risk factors	340	3.45 (3.03 – 3.73)	950	2.34 (2.17 – 2.52)

The *HR*s were adjusted for age, sex, race (not adjusted in CCDRFS), residence area (adjusted in CCDRFS only), education level, income, history of CVD and cancer, and diabetes duration (not adjusted in CCDRFS). *T2D* type 2 diabetes, *CVD* cardiovascular disease, *CCDRFS* China Chronic Disease and Risk Factors Surveillance, *NHANES* National Health and Nutrition Examination Survey

Table S3 Adjusted hazard ratios (*HR*s) for mortality by weighted risk scores among patients with T2D vs. controls without T2D

Cohorts	Subgroups	CVD mortality		All-cause mortality	
		**Death (*n*)**	***HR* (95% CI)**	**Death (*n*)**	***HR* (95% CI)**
CCDRFS					
	Matched controls	1365	Reference	2974	Reference
	People with T2D				
	Optimal	191	0.90 (0.67 – 1.20)	392	0.98 (0.82 – 1.17)
	Suboptimal	652	1.57 (1.21 – 2.03)	1498	1.60 (1.42 – 1.81)
	Poor	99	2.10 (1.36 – 3.26)	186	2.27 (1.71 – 3.01)
USA NHANES					
	Matched controls	743	Reference	2342	Reference
	People with T2D				
	Optimal	31	1.13 (0.78 – 1.64)	78	0.88 (0.61 – 1.25)
	Suboptimal	379	1.62 (1.38 – 1.90)	1069	1.45 (1.31 – 1.59)
	Poor	152	2.34 (1.85 – 2.96)	467	2.20 (1.90 – 2.54)
UK Biobank					
	Matched controls	884	Reference	4033	Reference
	People with T2D				
	Optimal	40	1.62 (1.18 – 2.22)	105	0.97 (0.80 – 1.18)
	Suboptimal	835	2.25 (2.04 – 2.47)	2628	1.66 (1.58 – 1.75)
	Poor	400	3.29 (2.91 – 3.72)	1227	2.48 (2.32 – 2.65)

*HR*s were adjusted for age, sex, race (not adjusted in CCDRFS), residence area (adjusted in CCDRFS only), education level, income, history of CVD and cancer, and diabetes duration (not adjusted in CCDRFS). *T2D* type 2 diabetes, *CVD* cardiovascular disease, *CCDRFS* China Chronic Disease and Risk Factors Surveillance, *NHANES* National Health and Nutrition Examination Survey

Table S4 Adjusted hazard ratios (*HR*s) for mortality by numbers of risk factors outside target ranges*, among patients with T2D vs. controls without T2D

Cohorts	Subgroups	CVD mortality		All-cause mortality	
		Death (*n*)	*HR* (95% CI)	Death (*n*)	*HR* (95% CI)
CCDRFS					
	Matched controls	1365	Reference	2969	Reference
	People with T2D				
	≤ 1 risk factor	77	0.93 (0.63 – 1.38)	180	1.13 (0.86 – 1.48)
	2 – 4 risk factors	764	1.44 (1.12 – 1.86)	1690	1.48 (1.31 – 1.68)
	≥ 5 risk factors	100	2.03 (1.36 – 3.04)	205	2.14 (1.67 – 2.74)
USA NHANES					
	Matched controls	540	Reference	1732	Reference
	People with T2D				
	≤ 1 risk factor	29	0.97 (0.65 – 1.45)	77	0.88 (0.67 – 1.17)
	2 – 4 risk factors	276	1.74 (1.43 – 2.12)	799	1.44 (1.30 – 1.60)
	≥ 5 risk factors	39	2.22 (1.41 – 3.49)	143	2.12 (1.62 – 2.77)
UK Biobank					
	Matched controls	884	Reference	4033	Reference
	People with T2D				
	≤ 1 risk factor	82	1.66 (1.33 – 2.08)	161	1.20 (1.09 – 1.32)
	2 – 4 risk factors	970	2.39 (2.18 – 2.62)	2910	1.78 (1.70 – 1.87)
	≥ 5 risk factors	223	3.62 (3.13 – 4.19)	889	2.43 (2.24 – 2.64)

*Using individual HbA1c target as the glycemic target and LDL-C levels as the lipid target. LDL-C values contained missing data in both CCDRFS and NHANES. *HR*s were adjusted for age, sex, race (not adjusted in CCDRFS), residence area (adjusted in CCDRFS only), education level, income, history of CVD and cancer, and diabetes duration (not adjusted in CCDRFS). *T2D* type 2 diabetes, *CVD* cardiovascular disease, *CCDRFS* China Chronic Disease and Risk Factors Surveillance, *NHANES* National Health and Nutrition Examination Survey, *LDL-C* low-density lipoprotein-cholesterol

Table S5 Adjusted hazard ratios (*HR*s) for mortality by numbers of risk factors outside target ranges among patients with T2D vs. all individuals without T2D

Cohorts	Subgroups	CVD mortality		All-cause mortality	
		Death (*n*)	*HR* (95% CI)	Death (*n*)	*HR* (95% CI)
CCDRFS					
	People without T2D	3146	Reference	7118	Reference
	People with T2D				
	≤ 1 risk factor	80	0.93 (0.65 – 1.35)	212	1.13 (0.88 – 1.46)
	2 – 4 risk factors	756	1.44 (1.16 – 1.78)	1669	1.47 (1.33 – 1.62)
	≥ 5 risk factors	106	2.00 (1.36 – 2.95)	195	2.07 (1.62 – 2.63)
USA NHANES					
	People without T2D	873	Reference	2894	Reference
	People with T2D				
	≤ 1 risk factor	25	1.02 (0.62 – 1.69)	66	0.83 (0.63 – 1.12)
	2 – 4 risk factors	396	1.54 (1.33 – 1.79)	1147	1.43 (1.30 – 1.56)
	≥ 5 risk factors	141	2.48 (1.99 – 3.11)	401	2.22 (1.91 – 2.58)
UK Biobank					
	People without T2D	4693	Reference	23,506	Reference
	People with T2D				
	≤ 1 risk factor	49	1.69 (1.28 – 2.24)	150	1.16 (0.99 – 1.37)
	2 – 4 risk factors	849	2.25 (2.09 – 2.43)	2773	1.72 (1.65 – 1.79)
	≥ 5 risk factors	377	3.42 (3.07 – 3.81)	1037	2.36 (2.22 – 2.52)

The *HR*s were adjusted for age, sex, race (not adjusted in CCDRFS), residence area (adjusted in CCDRFS only), education level, income, history of CVD and cancer, and diabetes duration (not adjusted in CCDRFS). *T2D* type 2 diabetes, *CVD* cardiovascular disease, *CCDRFS* China Chronic Disease and Risk Factors Surveillance, *NHANES* National Health and Nutrition Examination Survey

Table S6 Adjusted hazard ratios (*HR*s) for mortality by number of risk factors outside target ranges among patients with T2D vs. controls without T2D, with further adjustment for medication use

Cohorts	Subgroups	CVD mortality		All-cause mortality	
		Death (*n*)	*HR* (95% CI)	Death (*n*)	*HR* (95% CI)
CCDRFS					
	Matched controls	1365	Reference	2974	Reference
	People with T2D				
	≤ 1 risk factor	80	0.83 (0.54 – 1.28)	212	0.94 (0.73 – 1.22)
	2 – 4 risk factors	756	1.43 (1.09 – 1.87)	1669	1.41 (1.23 – 1.62)
	≥ 5 risk factors	106	2.44 (1.71 – 3.48)	195	1.92 (1.49 – 2.48)
USA NHANES					
	Matched controls	743	Reference	2342	Reference
	People with T2D				
	≤ 1 risk factor	25	0.98 (0.58 – 1.65)	66	0.82 (0.62 – 1.09)
	2 – 4 risk factors	396	1.44 (1.17 – 1.77)	1147	1.38 (1.22 – 1.57)
	≥ 5 risk factors	141	2.23 (1.69 – 2.93)	401	2.04 (1.70 – 2.45)
UK Biobank					
	Matched controls	884	Reference	4033	Reference
	People with T2D				
	≤ 1 risk factor	49	1.31 (0.97 – 1.75)	150	1.01 (0.85 – 1.19)
	2 – 4 risk factors	849	1.52 (1.34 – 1.72)	2773	1.40 (1.31 – 1.49)
	≥ 5 risk factors	377	2.90 (1.84 – 2.50)	1037	1.84 (1.69 – 2.01)

The *HR*s were adjusted for age, sex, race (not adjusted in CCDRFS), residence area (adjusted in CCDRFS only), education level, income, history of CVD and cancer, and diabetes duration (not adjusted in CCDRFS), diabetes medications, antihypertensive drugs, and lipid-lowering agents. *T2D* type 2 diabetes, *CVD* cardiovascular disease, *CCDRFS* China Chronic Disease and Risk Factors Surveillance, *NHANES* National Health and Nutrition Examination Survey

Table S7 Stratified analysis of adjusted hazard ratios (*HR*s) for all-cause mortality by the number of risk factors outside target ranges among patients with T2D vs. controls without T2D [*HR* (95% CI)]

**Stratified variable**	**Matched controls**	**No. of risk factors outside the target range among people with T2D**			$P_{interaction}$
		**≤ 1 risk factor**	**2 – 4 risk factors**	**≥ 5 risk factors**	
CCDRFS					
Diabetes medication					0.73
No	Reference	1.04 (0.79 – 1.38)	1.42 (1.22 – 1.66)	1.69 (1.26 – 2.28)	
Yes	Reference	0.83 (0.51 – 1.33)	1.76 (1.51 – 2.04)	2.56 (1.70 – 3.84)	
Complications					0.53
No	Reference	1.06 (0.81 – 1.37)	1.58 (1.37 – 1.81)	2.10 (1.49 – 2.96)	
Yes	Reference	0.70 (0.32 – 1.55)	1.23 (0.99 – 1.53)	1.87 (1.24 – 2.81)	
SES					0.77
Low	Reference	1.01 (0.75 – 1.34)	1.65 (1.43 – 1.90)	2.22 (1.61 – 3.05)	
High	Reference	0.95 (0.60 – 1.51)	1.19 (1.01 – 1.41)	1.66 (1.12 – 2.46)	
USA NHANES					
Age at diagnosis					0.23
≤ 50 years	Reference	0.83 (0.45 – 1.55)	2.06 (1.72 – 2.48)	3.64 (2.88 – 4.61)	
> 50 years	Reference	0.82 (0.60 – 1.14)	1.31 (1.17 – 1.46)	1.81 (1.53 – 2.14)	
Diabetes medication					0.30
None	Reference	0.85 (0.60 – 1.20)	1.29 (1.12 – 1.50)	2.28 (1.70 – 3.07)	
Only pills	Reference	0.64 (0.41 – 1.02)	1.37 (1.19 – 1.58)	2.19 (1.67 – .2.84)	
Insulin	Reference	1.46 (0.61 – 3.50)	2.28 (1.80 – 2.89)	2.68 (1.99 – 3.61)	
Complications					0.64
No	Reference	0.79 (0.51 – 1.23)	1.46 (1.22 – 1.75)	2.63 (2.05 – 3.38)	
Yes	Reference	0.95 (0.65 – 1.37)	1.55 (1.36 – 1.77)	2.27 (1.90 – 2.72)	
SES					0.32
Low	Reference	0.87 (0.62 – 1.21)	1.51 (1.35 – 1.68)	2.25 (1.94 – 2.60)	
High	Reference	0.99 (0.58 – 1.67)	1.64 (1.27 – 2.13)	3.30 (2.15 – 5.06)	
UK Biobank					
Age at diagnosis					0.25

Stratified variable	Matched controls	No. of risk factors outside the target range among people with T2D			$P_{interaction}$
		≤ 1 risk factor	2 – 4 risk factors	≥ 5 risk factors	
≤ 50 years	Reference	1.49 (1.11 – 1.98)	2.10 (1.91 – 2.32)	2.75 (2.41 – 3.14)	
> 50 years	Reference	1.09 (0.89 – 1.33)	1.59 (1.50 – 1.69)	2.23 (2.05 – 2.42)	
Diabetes medication					0.01
None	Reference	1.03 (0.80 – 1.34)	1.53 (1.41 – 1.65)	1.98 (1.75 – 2.24)	
Only pills	Reference	1.48 (1.16 – 1.87)	1.64 (1.52 – 1.77)	2.23 (2.00 – 2.48)	
Insulin	Reference	0.97 (0.60 – 1.54)	2.43 (2.17 – 2.72)	3.15 (2.74 – 3.63)	
Complications					0.82
No	Reference	1.16 (0.90 – 1.43)	1.65 (1.54 – 1.76)	2.31 (2.09 – 2.55)	
Yes	Reference	1.48 (1.14 – 1.93)	2.09 (1.90 – 2.29)	2.82 (2.51 – 3.15)	
SES					0.29
Low	Reference	1.23 (1.02 – 1.48)	1.72 (1.63 – 1.82)	2.38 (1.20 – 2.57)	
High	Reference	1.09 (0.75 – 1.58)	1.66 (1.42 – 1.94)	2.30 (1.89 – 2.80)	

The *HR*s were adjusted for age, sex, race (not adjusted in CCDRFS), residence area (adjusted in CCDRFS only), education level, income, history of CVD, cancer, and diabetes duration (not adjusted in CCDRFS). High socioeconomic status (SES) was defined in CCDRFS as meeting at least 2 of the following criteria: annual household income > 20,000 ¥, college education or higher, and urban residence; and in UK Biobank and NHANES as having income above the cohort-specific median and a college education or higher. Complications were defined in NHANES and UK Biobank as a history of myocardial infarction, stroke, retinopathy, or cancer, or estimated glomerular filtration rate < 60 ml/(min·1.73 m^2); and in CCDRFS as a history of myocardial infarction, stroke, or cancer. *T2D* type 2 diabetes, *CVD* cardiovascular disease, *CCDRFS* China Chronic Disease and Risk Factors Surveillance, *NHANES* National Health and Nutrition Examination Survey

Table S8 Adjusted hazard ratios (*HR*s) and years of life gained at age 50 by individual risk factors among patients with type 2 diabetes

Statistical measures	Physical activity [active vs. inactive (reference)]	Smoking [never vs. ever (reference)]	Diet [healthy vs. unhealthy (reference)]	Elevated HbA1c [yes vs. no (reference)]	Dyslipidemia [yes vs. no (reference)]	Elevated blood pressure [yes vs. no (reference)]
CCDRFS						
HR (95% CI)	0.69 (0.59 – 0.81)	0.77 (0.62 – 0.95)	0.92 (0.81 – 1.05)	0.64 (0.55 – 0.76)	0.95 (0.79 – 1.14)	0.93 (0.75 – 1.15)
Years of life gained (95% CI)						
Male	2.9 (1.8 – 4.1)	1.2 (1.0 – 1.5)	0.7 (-0.3 – 1.7)	3.5 (3.2 – 3.9)	0.3 (-1.7 to 2.5)	0.4 (-1.9 to 2.6)
Female	2.9 (1.8 – 4.1)	1.2 (1.0 – 1.6)	0.7 (-0.3 – 1.7)	3.5 (3.2 – 3.9)	0.3 (-1.7 to 2.3)	0.4 (-1.9 to 2.4)
US NHANES						
HR (95% CI)	0.65 (0.55 – 0.76)	0.74 (0.63 – 0.85)	0.84 (0.73 – 0.98)	0.83 (0.71 – 0.97)	1.06 (0.91 – 1.22)	0.87 (0.74 – 1.02)
Years of life gained (95% CI)						
Male	4.1 (3.8 – 4.3)	2.9 (2.6 – 3.2)	2.0 (1.5 – 2.3)	2.5 (2.2 – 2.8)	-0.1 (-0.4 – 0.2)	0.9 (0.7 – 1.1)
Female	3.7 (3.5 – 4.0)	2.7 (2.4 – 2.9)	1.8 (1.5 – 2.0)	2.3 (2.0 – 2.6)	-0.09 (-0.3 – 0.2)	0.8 (0.6 – 1.0)
UK Biobank						
HR (95% CI)	0.74 (0.70 – 0.79)	0.71 (0.66 – 0.76)	0.92 (0.85 – 1.00)	0.86 (0.81 – 0.92)	0.92 (0.85 – 0.99)	0.91 (0.85 – 0.97)
Years of life gained (95% CI)						
Male	2.7 (2.0 – 3.5)	2.7 (1.9 – 3.5)	0.8 (-0.2 to 1.7)	2.0 (1.3 – 2.6)	0.4 (-0.5 to 1.2)	0.7 (0 – 1.3)
Female	2.6 (1.8 – 3.3)	2.6 (1.8 – 3.3)	0.7 (0 – 1.6)	1.9 (1.3 – 2.6)	0.5 (-0.4 to 1.2)	0.7 (0 – 1.5)

The *HR*s applied to estimate life expectancy were adjusted for age, sex, race (not adjusted in CCDRFS), residence area (adjusted in CCDRFS only), education level, income, history of CVD and cancer, diabetes duration (not adjusted in CCDRFS), and each risk factor was adjusted for each other. *CCDRFS* China Chronic Disease and Risk Factors Surveillance, *NHANES* National Health and Nutrition Examination Survey

Table S9 Adjusted hazard ratios (*HR*s) for all-cause mortality according to genetic risk among participants with type 2 diabetes in the UK Biobank

Model	Low genetic risk	High genetic risk [*HR* (95% CI)]	*P*-value	Per SD increment in genetic risk [*HR* (95% CI)]
Model 1	Reference	1.07 (1.01 – 1.12)	0.03	1.04 (1.01 – 1.07)
Model 2	Reference	1.06 (1.01 – 1.13)	0.04	1.04 (1.01 – 1.07)

Model 1: adjusted for age, sex, and race. Model 2: further adjusted for education level, income, smoking status, physical activity, healthy diet, hypertension, dyslipidemia, CVD, cancer, genotype measurement batch, and genetic principal components. *T2D* type 2 diabetes, *CVD* cardiovascular disease

Figure legends

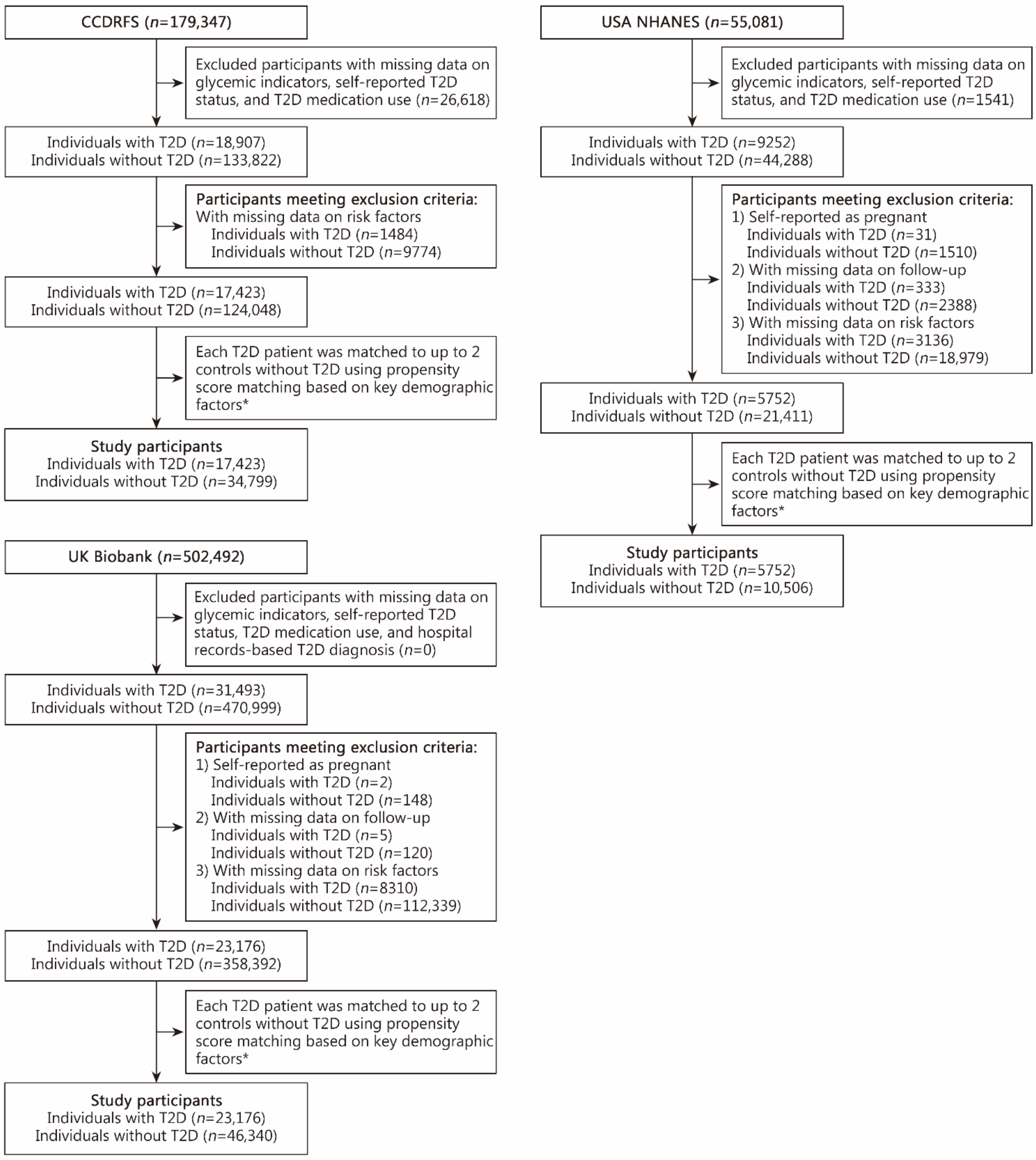

Fig. S1 Flow chart of study participants. This flowchart illustrates the selection of study participants from CCDRFS, USA NHANES, and UK Biobank. Individuals with missing information on diabetes status and glycemic indicators were first excluded. Among the remaining participants, those with missing data on key risk factors and other predefined exclusion criteria were further excluded. Finally, each individual with diabetes was matched to approximately 2 controls without diabetes and included in the analysis. *The matching criteria included age, sex, and residence area in the CCDRFS; age, sex, race, and survey cycles in USA NHANES; and age, sex, and race in UK Biobank. CCDRFS China Chronic Disease and Risk Factors Surveillance, NHANES National Health and Nutrition Examination Survey, T2D type 2 diabetes

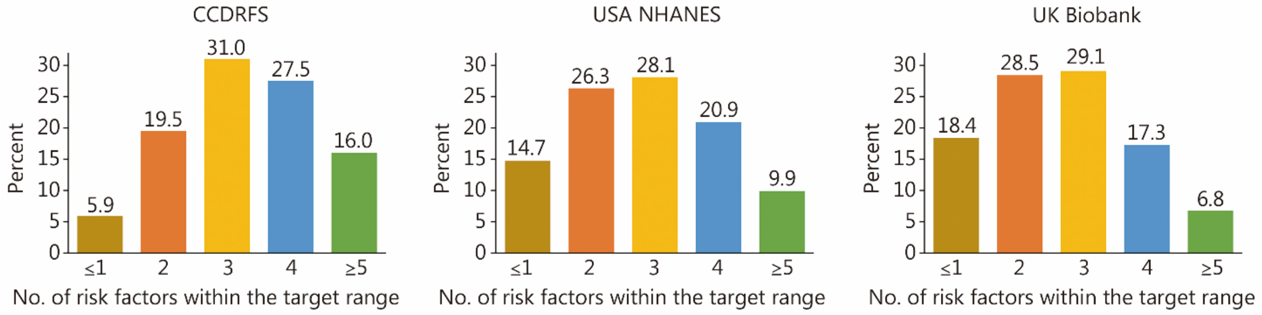

Fig. S2 Distribution of the number of risk factors within the target range in CCDRFS, USA NHANES, and UK Biobank cohorts. In CCDRFS and USA NHANES, all estimates accounted for complex survey designs. CCDRFS China Chronic Disease and Risk Factors Surveillance, NHANES National Health and Nutrition Examination Survey

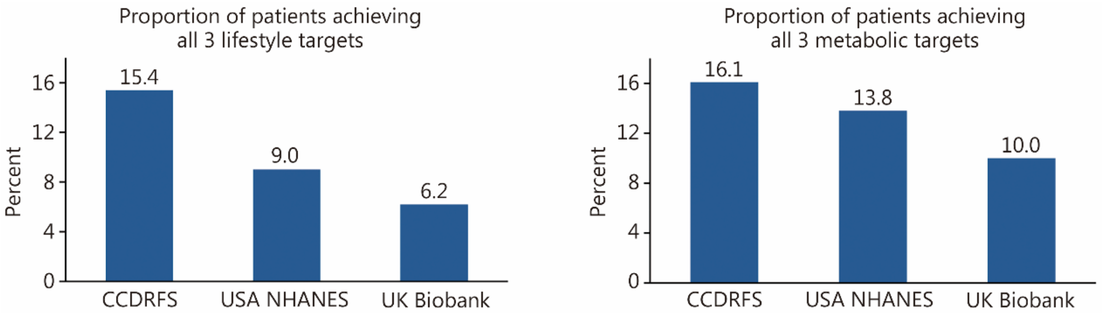

Fig. S3 Proportion of participants achieving all lifestyle targets and all clinical metabolic targets in CCDRFS, USA NHANES, and UK Biobank cohorts. In CCDRFS and USA NHANES, all estimates accounted for complex survey designs. CCDRFS China Chronic Disease and Risk Factors Surveillance, NHANES National Health and Nutrition Examination Survey

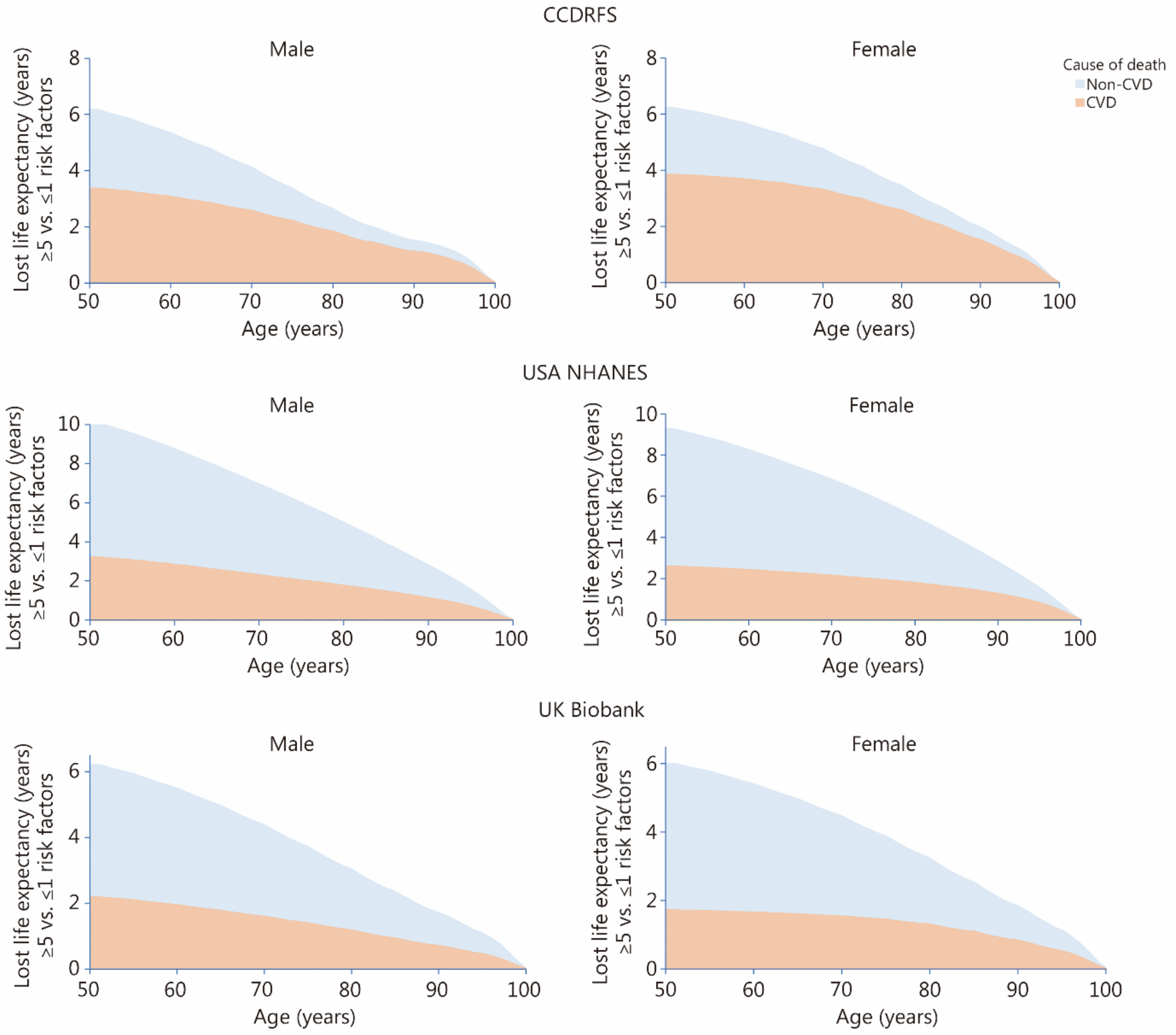

Fig. S4 Estimated years of life lost from having ≥ 5 risk factors vs. ≤ 1 risk factor attributable to reduced death from CVD and other causes among people with T2D. CVD cardiovascular disease, T2D type 2 diabetes, CCDRFS China Chronic Disease and Risk Factors Surveillance, NHANES National Health and Nutrition Examination Survey

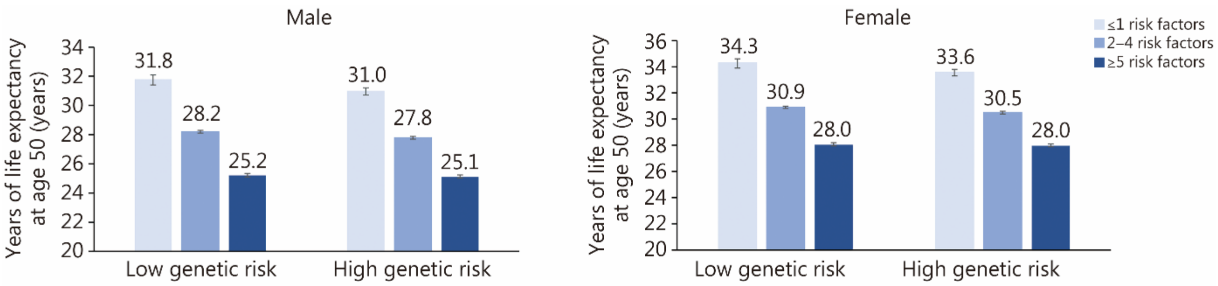

Fig. S5 Estimated life expectancy at age 50 according to the number of risk factors outside the target ranges in people with T2D stratified by genetic risk in the UK Biobank. The *HR*s applied to estimate life expectancy were adjusted for age, sex, race, education level, income, diabetes duration, history of CVD, cancer, genotype measurement batch, and genetic principal components. CVD cardiovascular disease, *HR*s hazard ratios, CCDRFS China Chronic Disease and Risk Factors Surveillance, NHANES National Health and Nutrition Examination Survey
